# Continuous Requirement for the Clr4 Complex But Not RNAi for Centromeric Heterochromatin Assembly in Fission Yeast Harboring a Disrupted RITS Complex

**DOI:** 10.1371/journal.pgen.1001174

**Published:** 2010-10-28

**Authors:** Sreenath Shanker, Godwin Job, Olivia L. George, Kevin M. Creamer, Alaa Shaban, Janet F. Partridge

**Affiliations:** 1 Department of Biochemistry, St. Jude Children's Research Hospital, Memphis, Tennessee, United States of America; 2 Integrated Program in Biomedical Sciences, University of Tennessee Health Science Center, Memphis, Tennessee, United States of America; University of California San Francisco, United States of America

## Abstract

Formation of centromeric heterochromatin in fission yeast requires the combined action of chromatin modifying enzymes and small RNAs derived from centromeric transcripts. Positive feedback mechanisms that link the RNAi pathway and the Clr4/Suv39h1 histone H3K9 methyltransferase complex (Clr-C) result in requirements for H3K9 methylation for full siRNA production and for siRNA production to achieve full histone methylation. Nonetheless, it has been proposed that the Argonaute protein, Ago1, is the key initial trigger for heterochromatin assembly via its association with Dicer-independent “priRNAs.” The RITS complex physically links Ago1 and the H3-K9me binding protein Chp1. Here we exploit an assay for heterochromatin assembly in which loss of silencing by deletion of RNAi or Clr-C components can be reversed by re-introduction of the deleted gene. We showed previously that a mutant version of the RITS complex (Tas3_WG_) that biochemically separates Ago1 from Chp1 and Tas3 proteins permits maintenance of heterochromatin, but prevents its formation when Clr4 is removed and re-introduced. Here we show that the block occurs with mutants in Clr-C, but not mutants in the RNAi pathway. Thus, Clr-C components, but not RNAi factors, play a more critical role in assembly when the integrity of RITS is disrupted. Consistent with previous reports, cells lacking Clr-C components completely lack H3K9me2 on centromeric DNA repeats, whereas RNAi pathway mutants accumulate low levels of H3K9me2. Further supporting the existence of RNAi–independent mechanisms for establishment of centromeric heterochromatin, overexpression of *clr4^+^* in *clr4Δago1Δ* cells results in some de novo H3K9me2 accumulation at centromeres. These findings and our observation that *ago1Δ* and *dcr1Δ* mutants display indistinguishable low levels of H3K9me2 (in contrast to a previous report) challenge the model that priRNAs trigger heterochromatin formation. Instead, our results indicate that RNAi cooperates with RNAi–independent factors in the assembly of heterochromatin.

## Author Summary

Centromeres are the chromosomal regions that promote chromosome movement during cell division. They consist of repetitive DNA sequences that are packaged into heterochromatin. Disruption of centromeric heterochromatin leads to chromosome loss that can result in miscarriages and genetic disorders. We have sought to define the precise steps leading to heterochromatin assembly using fission yeast as the model system. To accomplish this we employed our novel Tas3_WG_ mutant strain that can propagate preassembled heterochromatin but cannot support its de novo establishment. Current models suggest that small RNAs initiate heterochromatin assembly by targeting the RNAi machinery and subsequently the Clr-C chromatin-modifying complex to the centromere. Here, we demonstrate that transient depletion of components of the RNAi pathway that generate or bind small RNAs does not perturb heterochromatin assembly in our Tas3_WG_ strain. Instead, transient depletion of the Clr-C complex blocks heterochromatin assembly, suggesting a critical role for continuous Clr-C activity during heterochromatin assembly in Tas3_WG_ cells. We have directly tested whether Clr-C can target centromeres when expressed in cells deficient for RNAi and Clr-C. We find that RNAi–independent recruitment of Clr-C can occur and likely contributes to the critical initiating mechanisms of heterochromatin assembly.

## Introduction

Eukaryotic genomes are characterized by domains of transcriptionally permissive euchromatin and relatively transcriptionally inert heterochromatin. In addition to its important role in transcriptional regulation, heterochromatin plays a critical role in the regulation of genomic stability. In fission yeast constitutive heterochromatin assembles at the centromeres, telomeres and the mating type locus. This heterochromatin is required for high fidelity chromosome transmission, protecting chromosome ends from fusion to form dicentric chromosomes, and preventing co-expression of both sets of mating type information which could lead to haploid meiosis and cell death.

A major hallmark of heterochromatin in most eukaryotes is the presence of methyl groups on lysine 9 of histone H3. In fission yeast (*Schizosaccharomyces pombe*), methylation of H3 K9 is carried out by a single enzyme, Clr4 (the homolog of Suvar39 enzymes in higher eukaryotes), which is responsible for mono, di and tri-methylation of H3K9 [1]. This mark is in turn bound by proteins bearing a chromodomain, including the HP1 homologs Swi6 and Chp2, and importantly, Clr4 itself, leading to models for perpetuation and spreading of heterochromatin [2]–[5]. A fourth chromodomain protein, Chp1, has high affinity for binding the methyl mark [6]. Chp1 is a component of the RITS complex (RNA-induced initiation of transcriptional silencing complex), which is critical for the accumulation of heterochromatin at centromeres [7], [8].

Heterochromatin assembly in several organisms also depends upon the cellular RNA interference (RNAi) pathway [9]. RNAi is triggered by double-stranded RNA (dsRNA), which is processed by the RNAseIII-like activity of Dicer into short interfering (si) RNAs. siRNAs are loaded into RNA –induced silencing complexes (RISC), where they associate with Argonaute proteins, and base-pair with and promote the sequence-dependent destruction of RNA via cleavage mediated by Argonaute. In fission yeast, the RNAi effector complex is called RITS, and consists of the sole argonaute protein, Ago1, in complex with Tas3 which physically links Ago1 to Chp1 [8], [10]. Association of the RITS complex with centromeres is co-dependent on the RNA dependent RNA polymerase complex, RDRC [11]. RITS and RDRC physically associate with the Clr4-containing Clr-C complex [5], [12], and trigger an RNAi-mediated positive feedback loop to enhance H3K9me2 accumulation and heterochromatin assembly [13]. Accumulation of H3K9me2 allows recruitment of heterochromatin- binding proteins such as Swi6 (the fission yeast homolog of HP1) and cohesin to centromeric repeats, and is required for efficient chromosome segregation (reviewed in [14]). Clearly, the mechanism by which RITS and RDRC are initially recruited to centromeres to promote RNAi-dependent accumulation of H3K9me2 is critical to our understanding of heterochromatin assembly.

Somewhat paradoxically, the outer repeats of the centromere are transcribed by RNA polymerase II, and this transcription correlates with heterochromatin assembly [15]–[18]. Recently, two models have been proposed for how centromeric transcripts may initiate recruitment of RITS/RDRC. The first proposes that single stranded centromeric transcripts fold into hairpin structures to provide dsRNA template for the activity of dicer (Dcr1) to generate centromeric siRNAs to target RITS to homologous centromeric sequences [19]. Alternatively, RNA degradation products (priRNAs) associate with Ago1, and if derived from centromeric transcripts, target Ago1 to centromeres [20]. Ago1 slices transcripts that are homologous to priRNAs, recruiting RDRC which promotes dsRNA synthesis. dsRNAs are cleaved by Dcr1 to form centromeric siRNAs that recruit RITS to centromeres [8]. In both models, centromeric RITS/RDRC then promotes Clr-C association, and H3K9 methylation facilitating binding of Chp1 to chromatin.

These models infer that small RNAs and the RNAi pathway act as the priming signal for heterochromatin assembly, with Dcr1 or Ago1 playing the initiating role respectively. However, RITS possesses two potential centromeric targeting motifs: Ago1 which binds siRNAs and can target centromeric transcripts and Chp1 which has high affinity for binding H3K9me2 [6], [7]. We questioned whether indeed RNAi is the upstream event for heterochromatin assembly, or whether Clr4 functions upstream of RNAi to generate H3K9me2 to recruit RITS.

Dissection of the requirements for the initial assembly of centromeric heterochromatin is greatly hampered by the inter-relatedness of the RNAi and chromatin modifying pathways and the positive feedback loops involved in full heterochromatin assembly. Deletion of genes required for assembly of heterochromatin ablates heterochromatin, complicating analysis of whether the gene contributes to the establishment or maintenance of heterochromatin. To define the contribution of siRNAs and H3K9me to targeting RITS to centromeres, we have generated mutants that separate Ago1 from the RITS complex [10], that remove Chp1 from the RITS complex [21], and mutations within the chromodomain of Chp1 [6], [22] that weaken the high affinity of Chp1's chromodomain for binding H3K9me2/3. Data accumulated from analysis of these mutants strongly supports that centromeric targeting of RITS critically depends on Chp1 and in particular, Chp1 chromodomain's high affinity for binding H3K9me2/3.

The *tas3_WG_* mutant separates Ago1 from Tas3-Chp1 [10]. This mutant bears a two amino acid alanine substitution of residues W265 and G266 within the conserved WG/GW Ago “hook” (or interaction domain) of Tas3 [10], [23], that renders the Chp1-Tas3 subcomplex incapable of associating with Ago1. Surprisingly, *tas3_WG_* cells can maintain preassembled heterochromatin, most likely via retention of the subcomplexes of RITS at centromeres because of Chp1-Tas3 association with H3K9me2 and Ago1's association with centromeric siRNAs [10]. Interestingly, following removal of all H3K9me2 and loss of heterochromatin –dependent siRNAs (in *clr4Δ* backgrounds), *tas3_WG_* cells fail to generate de-novo heterochromatin on reintegration of *clr4^+^* [10]. We reasoned that genes that are particularly important for the establishment of heterochromatin would likewise be defective for heterochromatin assembly if transiently depleted in the *tas3_WG_* background. In contrast, genes with a less critical role for heterochromatin assembly might be expected to assemble heterochromatin efficiently if transiently depleted in *tas3_WG_* cells.

Here, we directly test the contribution of proteins involved in the RNAi pathway or in methylation of H3K9 to the initiation of centromeric heterochromatin. We find that transient depletion of any Clr-C component perturbs the establishment of heterochromatin in *tas3_WG_* cells. In contrast, transient depletion of genes involved in the RNAi pathway does not block heterochromatin assembly in *tas3_WG_* cells. Thus there appears to be a continuous requirement for the Clr-C complex, but not RNAi, during heterochromatin assembly in cells bearing a disrupted RITS complex. Consistent with this, RNAi-defective cells retain low levels of the heterochromatin mark, H3K9me2, whereas this mark is completely absent from Clr-C mutant cells. Because RNAi-defective cells maintain residual H3K9me2, it is not heterochromatin initiation which is being monitored following reintroduction of RNAi components, but more downstream aspects of heterochromatin assembly. To determine if RNAi is required for the initial step in heterochromatin assembly, we additionally removed H3K9me2 from RNAi defective cells by making compound mutants with *clr4Δ*, and interrogated whether on re-expression of *clr4^+^*, Clr-C could target centromeric sequences. We found that Clr4 can promote de novo methylation of centromeric repeats when overexpressed in cells otherwise lacking Clr4 and either of the RNAi components Ago1 or Dcr1. Thus, Clr-C can target H3K9me2 to centromeric repeats independently of the RNAi pathway. This data, plus our finding that *ago1*-deficient cells retain significant levels of H3K9me2 on centromeric repeats shows that Ago1 and Ago1 bound priRNAs are not necessary for the initiation of assembly of centromeric heterochromatin. Instead, our data strongly indicates that RNAi-independent mechanisms function together with RNAi in the cooperative assembly of centromeric heterochromatin.

## Results

### Transient depletion of *rik1^+^* causes defective heterochromatin establishment in *tas3_WG_* cells

To precisely define the point of action of regulators of heterochromatin assembly, we have employed the *tas3_WG_* allele which disrupts the RITS complex. Transient gene depletion experiments in *tas3_WG_* cells previously showed that the H3K9 methyltransferase, Clr4, is required to establish centromeric heterochromatin when Ago1 is separated from Chp1-Tas3 [10]. Clr4 is a component of a large complex of proteins called Clr-C. Cells lacking Clr-C components lose centromeric heterochromatin [24]–[29], but the role of individual Clr-C components in heterochromatin assembly is poorly understood. We therefore assessed the point of action of Clr-C components in heterochromatin establishment, using transient gene depletion experiments in the *tas3_WG_* background.

**Figure 1:**
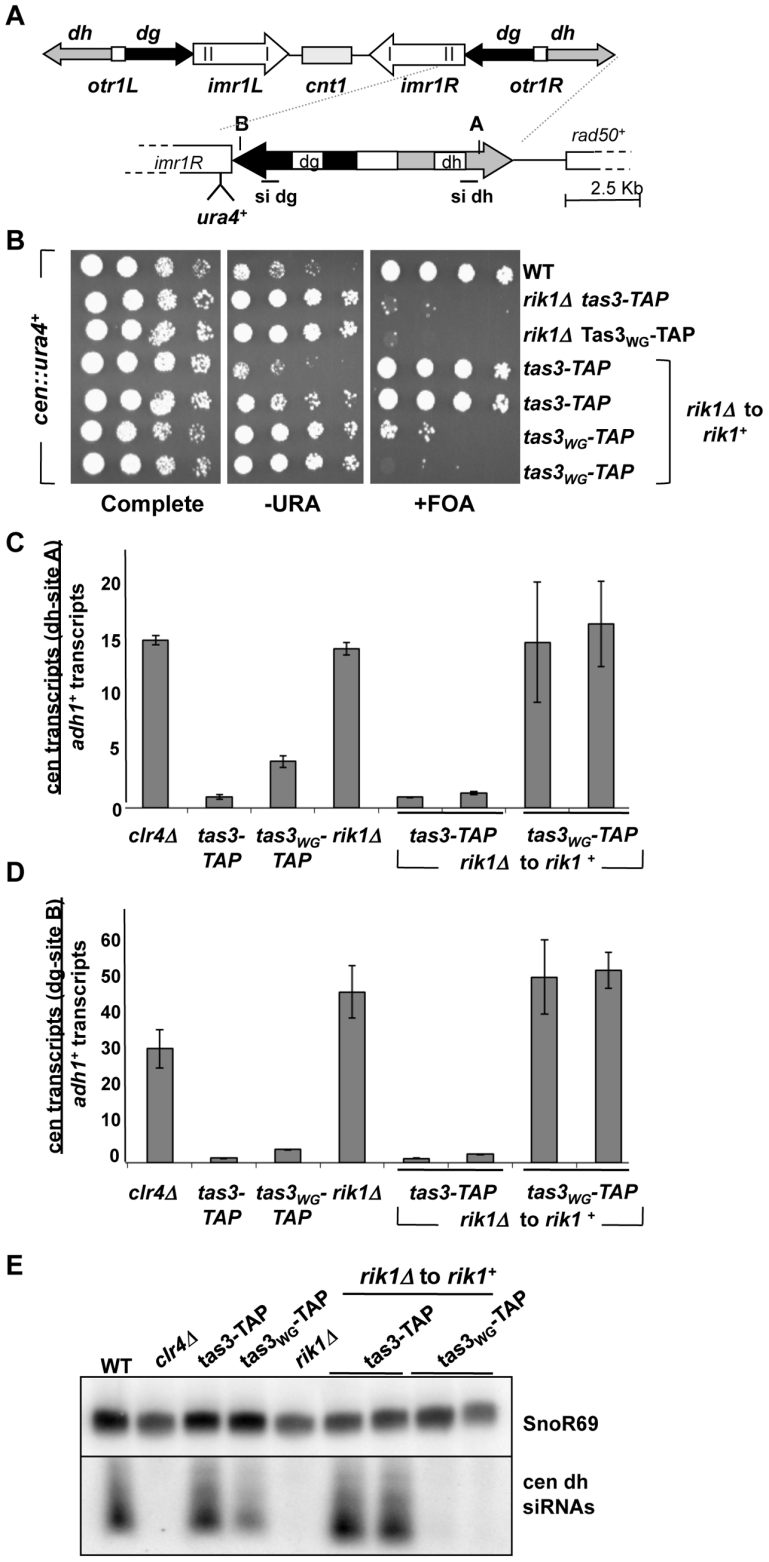
Clr-C component *rik1^+^* is required for establishment of repressive chromatin at the centromere. A. Cartoon representing the *dg* and *dh* repeat structure within the outer repetitive sequences (*otr*) at centromere 1. In the *cen::ura4^+^* strain, the *ura4^+^* marker gene is located within *dg*. A and B represent PCR fragments for real-time PCR analyses from *dh* and *dg* respectively, and probes used for siRNA detection are also represented. B. Serial dilution assay of yeast bearing the *cen::ura4^+^* marker plated onto complete synthetic media (PMG complete), media lacking uracil (−URA), or complete media supplemented with 5-fluoro-orotic acid (+FOA). In the last 4 rows, the *rik1^+^* gene has been reintegrated into the *rik1Δ* locus (*rik1Δ* to *rik1^+^*). Strains analyzed: PY2036, 3776, 3778, 4879, 4880, 4881, 4882. C. Real time PCR analysis of centromeric transcripts from the *dh* repeats (site A), relative to the *adh1^+^* euchromatic control in cDNA from indicated strains. Duplicate experiments used independent RNA samples, and data represents the mean ± SEM. All values were normalized to the value obtained for a wild type *cen::ura4^+^* strain (PY2036). Strains used: PY 2036, 1838, 3494, 3497, 3776, 4879, 4880, 4881, 4882. D. Real time PCR analysis performed as described for (C), but measuring the transcript accumulation from the *dg* repeats (site B) relative to *adh1^+^*. E. Northern blot of small RNA populations isolated from indicated strains, hybridized with probes for siRNAs derived from the *dh* repeat, or SnoR69 RNA as loading control. Strains used as in (C).

Rik1 was the first protein identified in complex with Clr4 [30], and it resembles the UV DNA damage binding protein, UV-DDB1, including homology to the CPSF-A factor involved in RNA processing [31]. Rik1 is thought to act upstream of Clr4, and to help recruit Clr-C to chromatin, since Rik1 remains localized at centromeres in mutants that mislocalize Clr4 [5], [28]. We tested whether transient depletion of *rik1* ^+^ in *tas3_WG_* cells would prevent assembly of heterochromatin. We introduced the *tas3_WG_-TAP* and *tas3-TAP* alleles into a *rik1Δ* strain that carries a *ura4* ^+^ transgene within the outer repeats of centromere 1 (*cen::ura4* ^+^). Wild type cells efficiently assemble heterochromatin on *cen::ura4* ^+^, silencing its expression, and allowing growth on media containing the drug 5-FOA, which is toxic to cells that express *ura4^+^*. Cells lacking *rik1^+^* fail to silence the centromeric transgene. The re-establishment of centromeric heterochromatin was monitored following reintroduction of *rik1^+^* into its normal genomic locus. Addition of *rik1^+^* to *rik1Δ tas3-TAP* cells allowed efficient establishment of heterochromatin and silencing of the *cen::ura4^+^* transgene (Figure 1B). In contrast, on reintroduction of *rik1^+^* into *tas3_WG_-TAP* cells, heterochromatin did not reassemble to silence the *cen::ura4^+^* reporter.

Transcription of endogenous *dg* and *dh* centromeric repeats was measured by real time PCR in cDNA prepared from these strains. In wild type cells, centromeric transcripts are processed by siRNA-dependent Ago1-mediated processing and by RNAi-independent turnover [32]–[34]. In addition, heterochromatin that assembles on repeat sequences can reduce access of RNA polymerase, thus preventing transcript accumulation [35]. Centromeric transcripts from *dh* (Figure 1C) and *dg* (Figure 1D) accumulate in cells lacking *rik1^+^*, similar to cells lacking *clr4^+^*. On reintegration of *rik1^+^* into *tas3-TAP* cells, centromeric transcripts become normally processed, resulting in no net gain in transcript levels in *rik1Δ* to *rik1^+^ tas3-TAP* cells relative to *tas3-TAP* cells. Strikingly, both *dg* and *dh* transcript levels remain high in *tas3_WG_* cells following reintegration of *rik1^+^*, consistent with the observed silencing defect of the *cen::ura4^+^* reporter in these strains. This accumulation of transcripts is at least in part due to defective processing of centromeric transcripts into siRNAs by the RNAi machinery since siRNAs were not detectable by Northern blotting in *tas3_WG_-TAP rik1Δ* to *rik1^+^* cells whereas *rik1^+^* reconstituted *tas3-TAP* cells synthesized centromeric siRNAs as efficiently as *tas3-TAP* cells (Figure 1E).

### Transient depletion of Raf1 or Raf2 in *tas3_WG_* cells causes defective heterochromatin assembly

Raf1 (Cmc1, Dos1, Clr8) and Raf2 (Cmc2, Dos2, Clr7) have also been identified as components of Clr-C [27], [29]. They are required for localization of Swi6 [25], and are important for silencing the mating type locus [26]. *raf1^+^* encodes a WD repeat protein which can bind Rik1 [25], and *raf2^+^* encodes a putative Zn finger protein which binds to Pcu4 [26].

**Figure 2:**
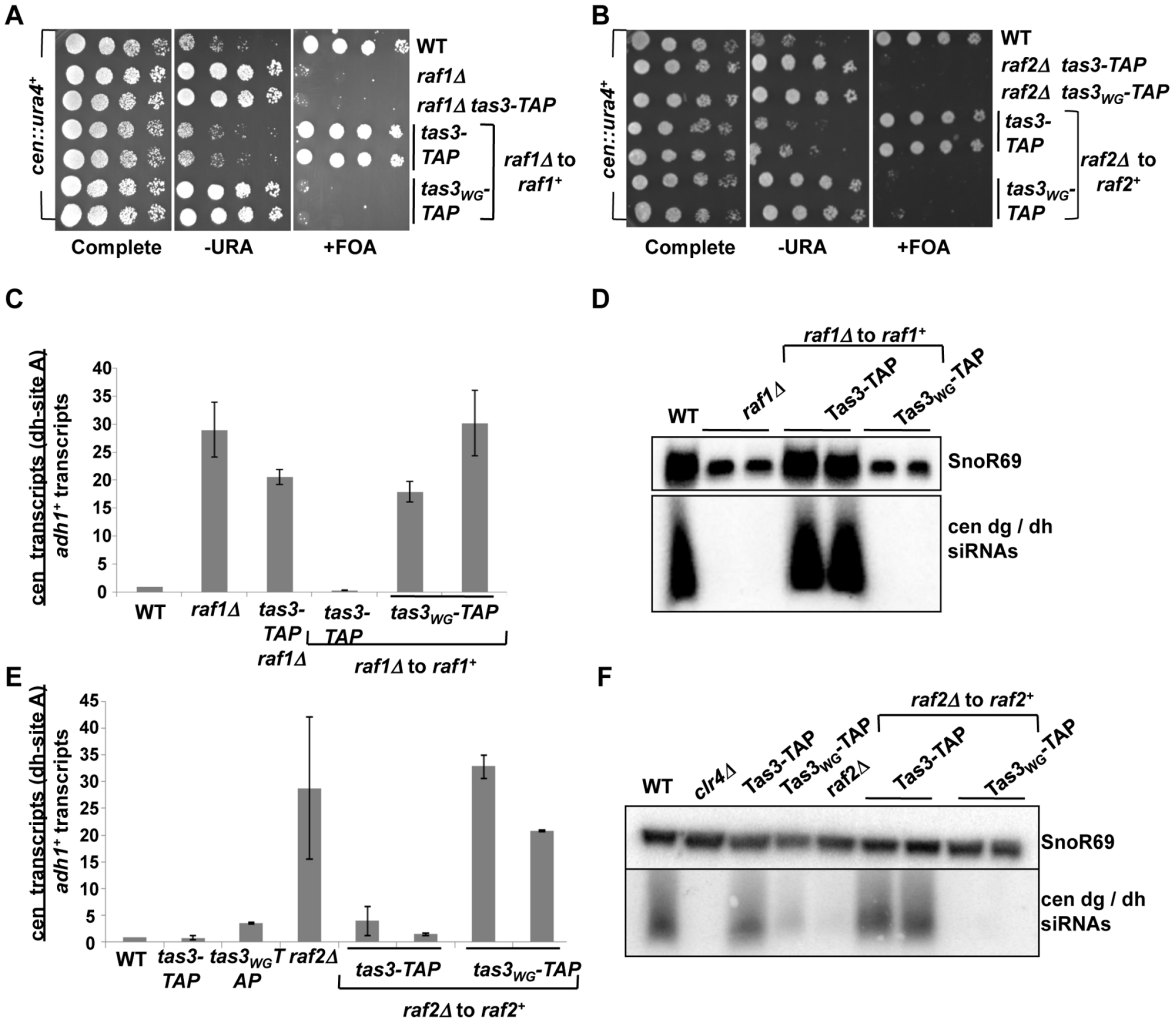
Clr-C components *raf1^+^* and *raf2^+^* facilitate establishment of repressive centromeric heterochromatin. A. Serial dilution assay of *raf1^+^* reintegration strains bearing the *cen::ura4^+^* reporter, and plated on specified media. Strains analyzed: PY2036, 3287, 3659, 3707, 3708, 3710, 3711. B. Serial dilution assay to monitor heterochromatin establishment in *raf2Δ* cells into which *raf2^+^* has been reintegrated in *tas3-TAP* and *tas3_WG_-TAP* backgrounds. Strains analyzed: PY2036, 3675, 3676, 3781, 3783, 3791, 3792. C. Real time PCR analysis of *dh* centromeric transcripts relative to transcripts from *adh1^+^*. Data shown represent the mean ± SEM measurements from cDNA derived from two independent cultures, normalized to the wild type *cen::ura4^+^* strain (PY2036). Strains analyzed: PY2036, 3287, 3659, 3707, 3710, 3711. See also Figure S1A. D. Northern blotting for *dg* and *dh* siRNAs and for the loading control SnoR69 on small RNA populations derived from indicated strains (as used in (C) and PY3708). E. Real time PCR analysis of cDNA to monitor centromeric transcript accumulation following reintegration of *raf2^+^* into *raf2Δ* cells bearing the *tas3-TAP* and *tas3_WG_-TAP* alleles. Transcripts from *dh* were measured relative to the *adh1^+^* control. Data were normalized to wild type *cen::ura4^+^* strains, and represents mean ± SEM. Strains analyzed: PY2036, 3494, 3497, 3293, 3781, 3783, 3791, 3792. See also Figure S1B. F. Northern blot analysis of siRNAs derived from *dg* and *dh* regions of the centromere, with SnoR69 as a loading control. Strains were as listed in (E).

*raf1Δ* and *raf2Δ* were crossed into *tas3-TAP* and *tas3_WG_* -TAP backgrounds, and wild type genomic copies of *raf1^+^* or *raf2^+^* were reintegrated into the corresponding deletion mutants and assessed for heterochromatin assembly. As seen for transient depletion experiments with *rik1*, *tas3-TAP* cells efficiently re-assembled centromeric heterochromatin on reintroduction of *raf1^+^* or *raf2^+^*, whereas silencing of the *cen::ura4^+^* reporter was not apparent in *tas3_WG_*-*TAP* backgrounds (Figure 2A and 2B). Centromeric transcripts accumulate to high levels in *raf1* and *raf2* deleted cells, and although transcript levels drop following reintegration of *raf1* ^+^ into *raf1Δ tas3-TAP* cells or of *raf2* ^+^ into *raf2Δ tas3-TAP* cells, high levels of *dg* and *dh* transcripts are maintained in both *raf1^+^* and *raf2^+^* reconstituted *tas3_WG_-TAP* cells (Figure 2C and 2E, Figure S1A and S1B). Consistent with this failure to suppress high levels of centromeric transcription in *tas3_WG_*-TAP cells transiently depleted for *raf1^+^* or *raf2^+^*, these cells fail to engage the RNAi pathway to promote destruction of centromeric transcripts into siRNAs (Figure 2D and 2F).

### Transient depletion of Pcu4 in *tas3_WG_* cells causes defective heterochromatin assembly

**Figure 3:**
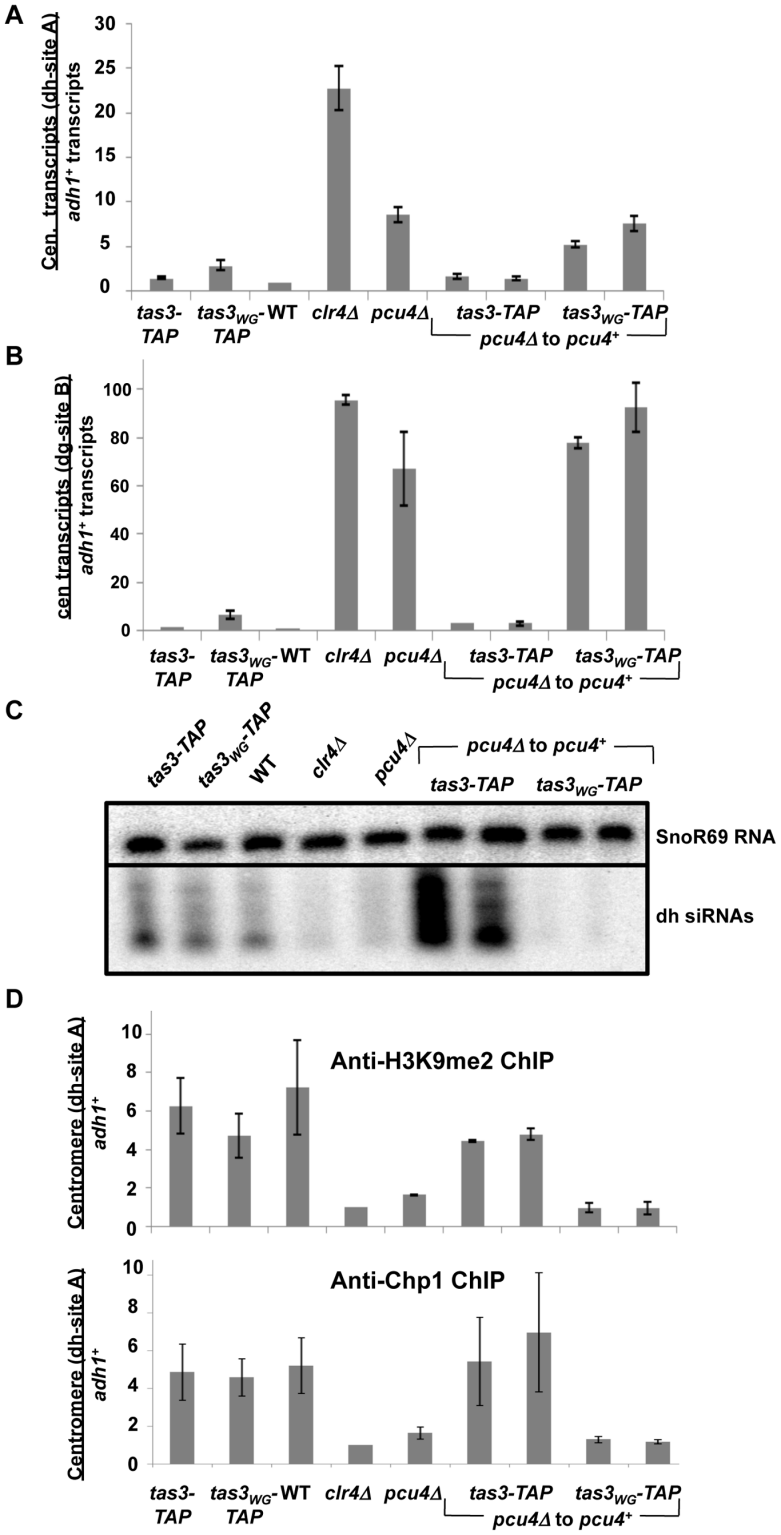
Pcu4 is required for establishment of repressive centromeric heterochromatin. A. Real time PCR analysis of centromeric transcripts from *dh* sequences relative to *adh1^+^* in cDNA derived from the indicated strains. Data represent mean ± SEM, normalized to wild type strain (PY41). Strains used were PY1065, PY2268, PY41, PY1797, PY3515, PY5082, PY5083, PY5085, PY5086. B. Real time PCR analysis of centromeric transcript accumulation from *dg* repeats. Samples were processed as described in (A). C. Northern blotting for *dh* siRNAs and SnoR69 as a loading control in small RNAs from strains listed in (A). D. ChIPs of H3K9me2 (upper panel) and Chp1 (lower panel) association with centromeric *dh* sequences, with *adh1* serving as control. Strains analyzed were as for (A), and data represents mean ± SEM from duplicate ChIP experiments, quantified by real time PCR.

Pcu4 is the fission yeast cullin4, and it has been identified in complex with the UV-DDB1 E3 ubiquitin ligase [35], [36], and with the related Rik1 protein in the Clr-C complex [27]–[29]. To define the role of Pcu4 in heterochromatin establishment, we monitored heterochromatin assembly following reintroduction of the *pcu4^+^* gene into *pcu4Δ tas3-TAP* and *tas3_WG_-TAP* strains. Clearly centromeric transcripts accumulate in *pcu4Δ* cells, and processing of transcripts is efficiently resumed following re-introduction of the wild type gene into *tas3-TAP* cells (Figure 3A and 3B). However, both *dh* and *dg* transcript levels are maintained at high levels following *pcu4^+^* reintroduction into *pcu4Δ tas3_WG_ -TAP* cells. This failure to silence centromeric transcripts was reflected in the failure to produce abundant centromeric siRNAs in these *tas3_WG_* reconstituted cells (Figure 3C).

In summary, all components of Clr-C are defective for silencing of endogenous centromeric or centromeric reporter transcripts following their transient depletion in *tas3_WG_* cells, suggesting that their continuous presence is required for the initiation of heterochromatin in RITS-defective cells.

### Defective heterochromatin assembly on transient depletion of Clr-C complex in *tas3_WG_* cells correlates with absence of centromeric H3K9me2

Next we analyzed H3K9 methylation on centromeric sequences following transient depletion of components of the Clr-C complex. In wild type cells, H3K9me accumulates to high levels on centromeric repeats through both RNAi-dependent and RNAi-independent mechanisms [15], [30]. In cells lacking *pcu4*, H3K9me2 levels on *dh* sequences are not above the background seen for *clr4Δ* cells which lack H3K9me2 (Figure 3D, upper panel). Following *pcu4* ^+^ reintroduction into *pcu4Δ tas3-TAP* cells, H3K9me2 levels rise to that seen in *tas3-TAP* cells, whereas no significant accumulation of H3K9me2 is observed in *pcu4* ^+^ reconstituted *tas3_WG_*-TAP cells. Thus Clr4 mediated H3K9 methylation is abrogated in *tas3_WG_-TAP* cells transiently depleted for *pcu4*. This methylation defect is not likely due to defective reassembly of the Clr-C complex following transient depletion of *pcu4*, since H3K9 methylation resumes effectively in *pcu4^+^* reconstituted *tas3-TAP* cells.

Chp1 binds H3K9me2/3 and Chp1 recruitment to centromeres is a hallmark of heterochromatin. ChIP experiments performed with anti-Chp1 antibodies demonstrated that *pcu4Δ* cells are also defective for Chp1 association with centromeres, and *pcu4^+^* reconstitution of *pcu4Δ tas3-TAP* but not of *pcu4Δ tas3_WG_* *-TAP* cells promotes Chp1 association with centromeres (Figure 3D, lower panel).

**Figure 4:**
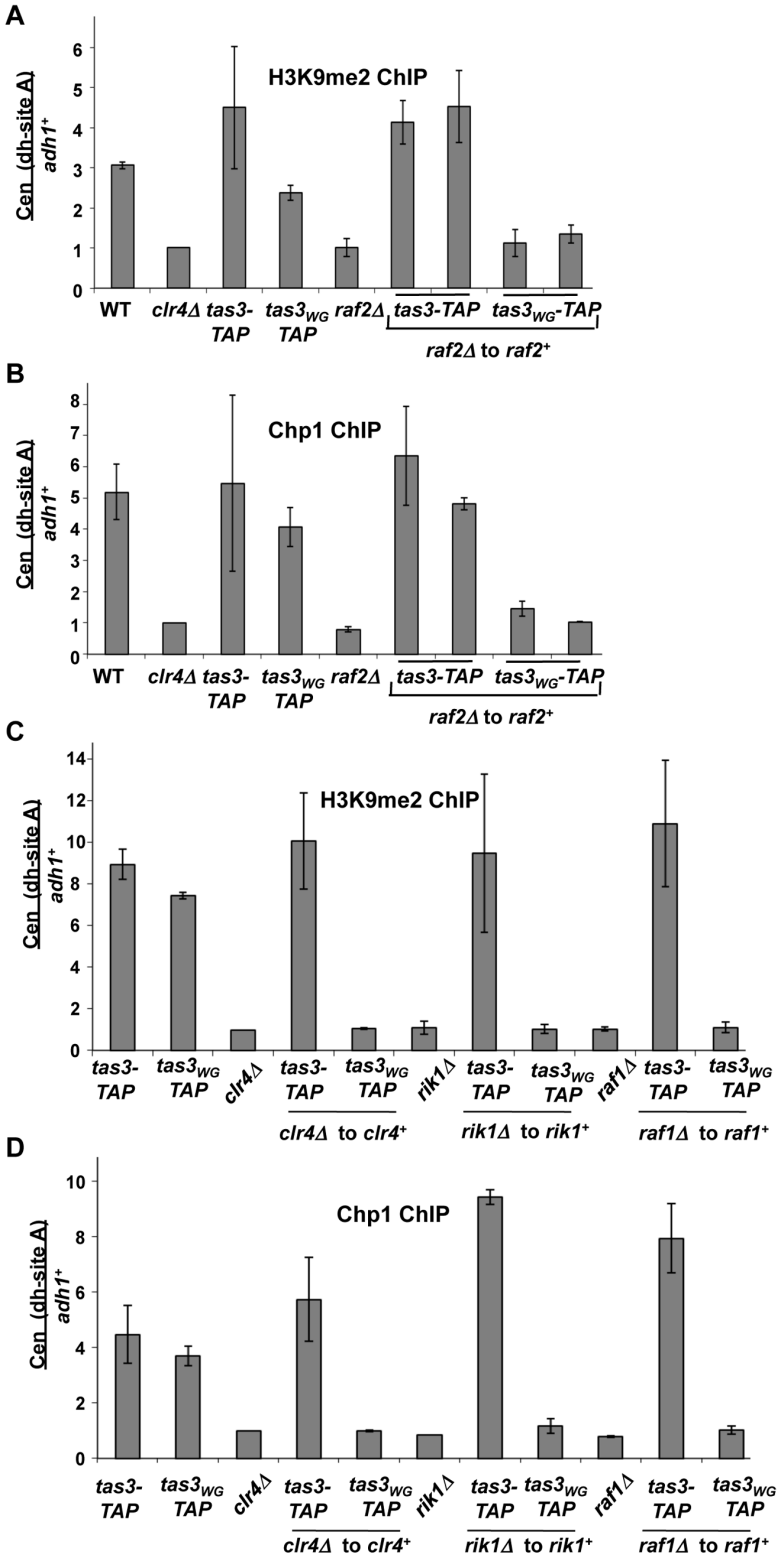
Components of the Clr-C complex are required for establishment of H3K9me2 at centromeres. A. ChIP analysis of H3K9me2 at *dh* repeats compared with *adh1^+^* locus measured by real time PCR. Enrichment of H3K9me2 was normalized to a strain lacking *clr4^+^*. Data represents the mean of 2 independent ChIP experiments ± SEM. Strains analyzed PY2036, 1838, 3494, 3497, 3293, 3781, 3783, 3791, 3792. B. ChIP analysis of Chp1 occupancy on centromeric *dh* repeats relative to *adh1^+^*, measured by real time PCR. ChIP values were normalized to strains lacking *clr4^+^*, and are the mean ± SEM of two independent ChIP experiments. Strains analyzed as in (A). C. Real time PCR quantitation of ChIP of H3K9me2 at centromeric *dh* repeats in *tas3-TAP* and *tas3_WG_-TAP* strains following reintegration of *clr4^+^*, *raf1^+^*, and *rik1^+^* into knockout backgrounds, measured relative to *adh1^+^*. Data represents mean ± SEM of 2 independent ChIP experiments and combine analyses for 2 reintegrants. Data were normalized to a strain lacking *clr4Δ* (PY1838). Strains analyzed PY3494, 3497, 1838, 2678, 2679, 2680, 2681, 3776, 4879, 4880, 4881, 4882, 3287, 3707, 3708, 3710, 3711. D. Real time PCR analysis of ChIP material derived from strains analyzed in (C), assessed for Chp1 association with centromeric *dh* repeats (site A). Data were processed as described in (C).

We also assessed H3K9me2 levels at centromeres in other strain backgrounds. In all Clr-C mutants (*raf2*, *rik1*, *raf1*), centromeric H3K9me2 levels were no higher than seen in *clr4Δ* cells (Figure 4A and 4C). Following reconstitution with *raf2^+^*, *clr4^+^*, *rik1^+^*, or *raf1^+^*, *tas3-TAP* cells accumulated “wild type” levels of H3K9me2, but *tas3_WG_*-TAP cells failed to accumulate H3K9me2 at centromeres.

Very similar results were obtained for Chp1 association with centromeres (Figure 4B and 4D), consistent with *tas3_WG_-TAP* cells being dependent on constitutive expression of all components of the Clr-C complex to provide H3K9me2 at centromeric sites for recruitment of Chp1. In sum, these experiments demonstrate that in cells where the association of Ago1 with Chp1-Tas3 has been abrogated, that reintroduction of Clr-C components is not sufficient to direct H3K9me2 accumulation on centromeric repeats. Clr-C defective cells should still express heterochromatin independent siRNAs, and Ago1 in these cells would be expected to maintain association with primal RNAs. Thus targeting of Ago1 by priRNAs to centromeric repeats is not sufficient to drive Clr-C recruitment to centromeres when Ago1 is physically separated from Tas3-Chp1.

### Dicer knockout cells show efficient assembly of centromeric heterochromatin on reintegration of the *dcr1* ^+^ gene

Next we examined whether RNAi components contribute to the initial steps in heterochromatin assembly. RNAi defective cells, such as *dcr1Δ*, are expected to retain priRNAs but lose most of their siRNAs. In contrast to Clr-C defective cells, *dcr1Δ* cells maintain low levels of H3K9me2 at centromeres (Figure S2D). Following overexpression of *dcr1^+^*, both *dcr1Δ tas3-TAP* and *dcr1Δ tas3_WG_ -TAP* cells efficiently assembled heterochromatin [10]. This suggested that H3K9me2, and not siRNA, acts at an early stage of heterochromatin initiation. However, in these experiments it was unclear whether overexpression of *dcr1^+^* suppressed an establishment defect in *tas3_WG_ dcr1^+^* reconstituted cells [10], [14].

We directly tested whether integration of *dcr1^+^* into the genomic *dcr1Δ* locus of *tas3-TAP* and *tas3_WG_-TAP* cells could support reassembly of centromeric heterochromatin. Cells lacking *dcr1^+^* fail to silence the *cen::ura4^+^* centromeric transgene. Following reintegration of *dcr1^+^*, silencing of the *cen::ura4* ^+^ reporter resumed in both *dcr1Δ* to *dcr1^+^ tas3-TAP* and *dcr1Δ* to *dcr1^+^ tas3_WG_*-*TAP* cells (Figure S2A). Cells lacking *dcr1^+^* accumulate high levels of centromeric transcripts, but following *dcr1^+^* reintegration, centromeric transcript levels were reduced in both the *dcr1^+^* reconstituted *tas3-TAP* and *tas3_WG_-TAP* cells, confirming that reintegration of *dcr1^+^* promoted efficient assembly of centromeric heterochromatin (Figure S2B, S2C). In addition, *dcr1Δ* cells cannot generate siRNAs from centromeric transcripts, but on reintegration of *dcr1^+^*, siRNA production resumed efficiently in both *tas3-TAP* and *tas3_WG_*-*TAP* backgrounds (Figure S2D). Together, these results confirmed and extended our data obtained with overexpressed *dcr1^+^* [10]. *dcr1^+^* and siRNAs are not critical for Clr-C activity at centromeres, but are important for amplification of the H3K9me2 signal during later stages of heterochromatin assembly.

### Components of the RDRC complex are not required for heterochromatin assembly in *tas3_WG_* cells

We next asked whether transient depletion of genes that act upstream of Dcr1 in the RNAi pathway would cause defective heterochromatin establishment. RDRC acts upstream of Dcr1, generating dsRNA for siRNA production. RDRC consists of the RNA-dependent RNA polymerase (Rdp1), the RNA helicase Hrr1, and a non-canonical poly (A) polymerase, Cid12 [11]. Cells lacking any component of RDRC show reduced RITS association and reduced H3K9me2 at centromeres, and have reduced siRNA production [11], [19], [20].

**Figure 5:**
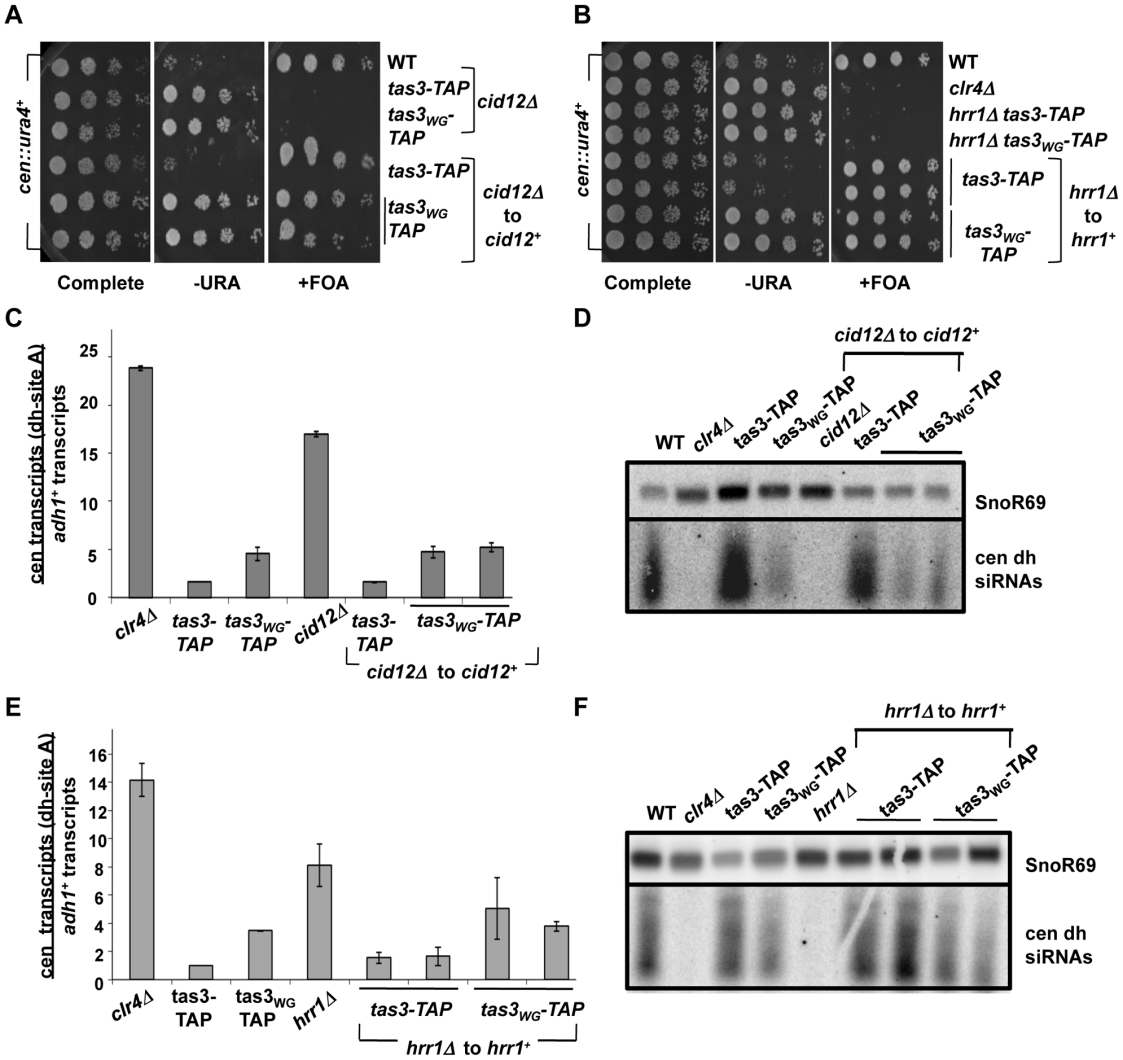
*cid12^+^* and *hrr1^+^* RDRC components are not required to initiate silencing of centromeric heterochromatin. A. Serial dilution spotting assay to monitor the role of *cid12^+^* in the silencing of the *cen::ura4^+^* reporter gene. Cells of the indicated genotypes and carrying the *cen::ura4^+^* reporter were plated onto selective media. Following reintegration of *cid12^+^* into the *cid12Δ* locus (*cid12Δ* to *cid12^+^*), both *tas3-TAP* and *tas3_WG_-TAP* cells grew on FOA media. Strains analyzed were PY2036, 4338, 4340, 4423, 4424, 4425. B. Serial dilution spotting assay of strains carrying the *cen::ura4^+^* reporter, following *hrr1^+^* reintegration at the *hrr1Δ* locus (*hrr1Δ* to *hrr1^+^*) and assessed for *ura4* expression compared with control strains on selective media. Strains analyzed: PY2036, 1838, 4334, 4336, 4420, 4481, 4586, 4587. C. Real time PCR analysis of cDNAs derived from indicated strains, quantifying centromeric *dh* transcripts relative to *adh1^+^* and normalized to ratio obtained in wild type cells. Data represent mean ± SEM from 2 independent RNA preparations. Strains analyzed: PY2036,1838, 3494, 3497, 4312, 4423, 4424, 4425. See also Figure S3A. D. Small RNA northern blot was hybridized with a probe for centromeric *dh* siRNAs, and with a probe for snoR69 as a loading control. Small RNAs were derived from strains used in (C). E. Real time PCR analysis of *dh* relative to *adh1^+^* transcripts in cDNA derived from indicated strains. Values represent means of 2 RNA preparations ± SEM. Strains analyzed: PY2036, 1838, 3494, 3497, 4308, 4420, 4481, 4586, 4587. See also Figure S3B. F. Northern blot for small RNA species hybridizing to the cen *dh* probe and to snoR69 as loading control. Strains are listed in (E).

**Figure 6:**
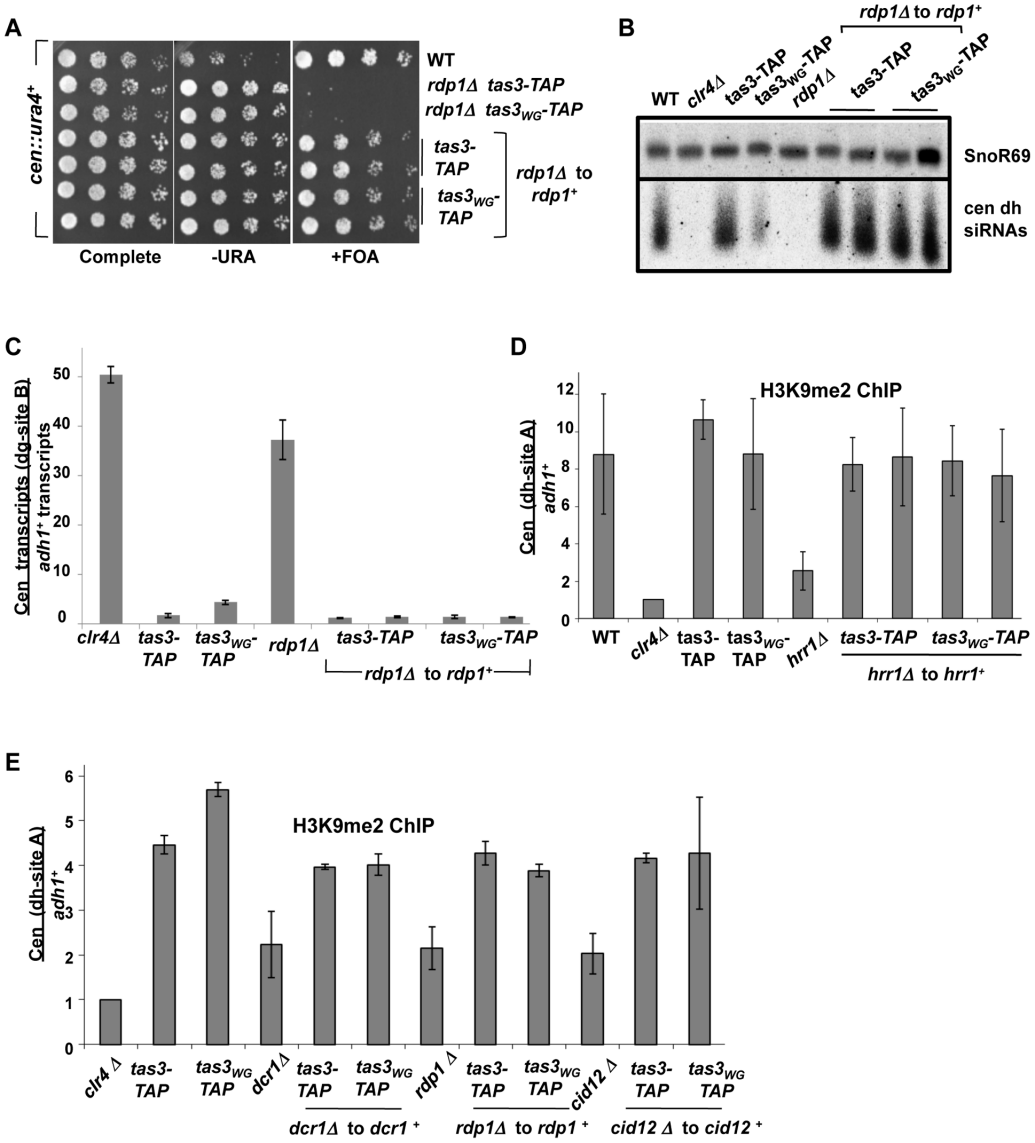
RDRC components are not required for establishment of silent H3K9me2 chromatin at centromeres. A. Serial dilution assay of strains bearing the *cen::ura4^+^* reporter and of the indicated genotype, plated on selective media. Strains analyzed: PY2036, 4300, 4304, 4401, 4402, 4403 and 4404. B. Northern blotting for small RNAs derived from *dh* centromeric repeats, with snoR69 RNA as loading control. Strains analyzed: PY 2036, PY1838, 3494, 3497, 4274, 4401, 4402, 4403, 4404. C. Real time PCR analysis of cDNA derived from strains of the indicated genotypes (as for Figure 6B), measuring centromeric *dh* transcripts normalized to *adh1^+^*, and to a wild type strain (PY2036). Data represents the mean ± SEM from analysis of 2 independent cDNA preparations from duplicate biological samples. See also Figure S4A. D. Real time PCR analysis of ChIP material derived using antibodies against H3K9me2, analyzed for centromeric *dh* sequences and normalized to euchromatic *adh1^+^* sequences. Data represent mean from two independent experiments ± SEM, normalized to data obtained from *clr4Δ* cells. Strains analyzed: PY2036, 1838, 3494, 3497, 4308, 4420, 4481, 4586, 4587. See also Figure S4B. E. ChIP for H3K9me2 at centromeric *dh* sequences relative to *adh1^+^*, normalized to a *clr4Δ* strain. Duplicate ChiP experiments were performed and data were processed as described above. Strains analyzed included *dcr1^+^* reintegration strains (*dcr1Δ* to *dcr1^+^*), *rdp1^+^* reintegration strains (*rdp1Δ* to *rdp1^+^*) and *cid12^+^* reintegrants (*cid12Δ* to *cid12^+^*). Two independent gene reintegrants for each *tas3_WG_-TAP* strain background were tested in duplicate. Strains analyzed: PY 2036, 3494, 3497, 3235, 3501, 3502, 3499, 3500, 4274, 4401, 4402, 4403, 4404, 4312, 4423, 4424, 4425.

We introduced the *tas3-TAP* and *tas3_WG_-TAP* alleles into deletion mutants of all components of RDRC, and then tested whether silenced chromatin assembled on the *cen::ura4^+^* reporter following reintegration of genomic clones encoding these genes. For cells lacking *cid12^+^*, *hrr1^+^*, or *rdp1^+^*, reintegration of these genes into knockout *tas3-TAP* cells allowed efficient assembly of heterochromatin. Interestingly, as seen for Dcr1, reintroduction of the genes into the mutant *tas3_WG_-TAP* strains also supported silencing of the *cen::ura4^+^* reporter (Figure 5A and 5B, Figure 6A).

Transcript analyses performed on the RDRC reconstituted strains revealed that cells lacking RDRC components accumulate both centromeric *dg* and *dh* transcripts, but that on reconstitution with the wild type gene, *dg* and *dh* transcript levels dropped to levels close to those normally found in *tas3-TAP* or *tas3_WG_-TAP* cells, which is considerably less than seen in RDRC mutant cells (Figure 5C and 5E, Figure 6C, and Figures S3A, S3B, and S4A). Thus processing of centromeric transcripts is efficiently resumed following reintroduction of RDRC components into RDRC deficient *tas3_WG_* cells, and this conclusion is further supported by detection of siRNAs in RDRC reconstituted cells (Figure 5D, 5F and Figure 6B). In summary, in contrast to cells transiently depleted for Clr-C components, centromeric heterochromatin assembly can occur efficiently following the transient depletion of RDRC components or of *dcr1* ^+^ in *tas3_WG_* cells.

### Low levels of H3K9me2 at centromeres in RDRC deficient cells support assembly of heterochromatin in reconstituted tas3WG cells

Cells lacking *dcr1^+^* accumulate H3K9me2 on centromeric sequences, whereas Clr-C deficient cells completely lack H3K9me2. We therefore asked whether cells lacking RDRC components accumulate centromeric H3K9me2, and whether H3K9me2 levels could signal the difference in outcome, promoting heterochromatin assembly in *tas3_WG_* cells following transient depletion of RDRC, but not Clr-C components.

We assessed centromeric H3K9me2 levels in RDRC deficient cells and following reintegration of RDRC components. In these experiments, we note that in all RDRC mutants, the level of H3K9me2 at centromeres is considerably higher than seen in cells lacking *clr4^+^*, although at least 2 fold reduced compared with wild type cells. Reintegration of RDRC components into the corresponding RDRC null cells supported centromeric accumulation of H3K9me2 of both *tas3-TAP* and *tas3_WG_-TAP* cells to levels found normally (Figure 6D and 6E). In addition, although Chp1 association with centromeres is diminished in *hrr1Δ* cells, reintroduction of *hrr1^+^* into *tas3_WG_-TAP* cells promoted Chp1 association (Figure S4B). Together then this data shows that heterochromatin assembly occurs efficiently following transient depletion of genes required for siRNA synthesis, including RDRC components that act upstream of Dcr1. In addition, the ability of heterochromatin to reform efficiently, following transient depletion of RNAi components in *tas3_WG_* cells, correlates with the persistence of low levels of H3K9me2 on centromeric repeats in the mutant backgrounds.

### Transient depletion of Ago1 in Tas3_WG_ cells causes no defect in heterochromatin establishment

Very recently it has been proposed that Ago1 is the most upstream factor in heterochromatin assembly. It is thought to act as an acceptor for RNA degradation products, termed pri-RNAs, which, based on frequency of occurrence, preferentially target antisense transcripts resulting from bidirectional transcription of DNA repeats. Cleavage of nascent centromeric transcripts by priRNA-directed activity of Ago1 is proposed to recruit the RDRC complex and eventually promote siRNA-dependent recruitment of RITS, and subsequent robust assembly of heterochromatin via recruitment of Clr-C [20]. This model therefore places Ago1 as an initiator, upstream of RDRC and Dcr1 and of the Clr-C complex and H3 K9 methylation. This model is supported by the detection of small RNAs (priRNAs) in *dcr1Δ* strains, and of siRNAs in cells lacking *clr4^+^*, or in which heterochromatin assembly is blocked because of mutation of H3K9, supporting that heterochromatin is not essential for small RNA generation [19], [20]. Finally, the model would suggest that siRNAs and priRNAs act upstream of heterochromatin assembly, and that Ago1 is the most upstream component of the RNAi pathway. Indeed, Halic and Moazed argue that Ago1 activity is required for the initial deposition of H3K9me, since in their publication strains lacking Ago1 exhibit lower levels of centromeric H3K9me accumulation than strains deficient in other components of the RNAi pathway [20].

**Figure 7:**
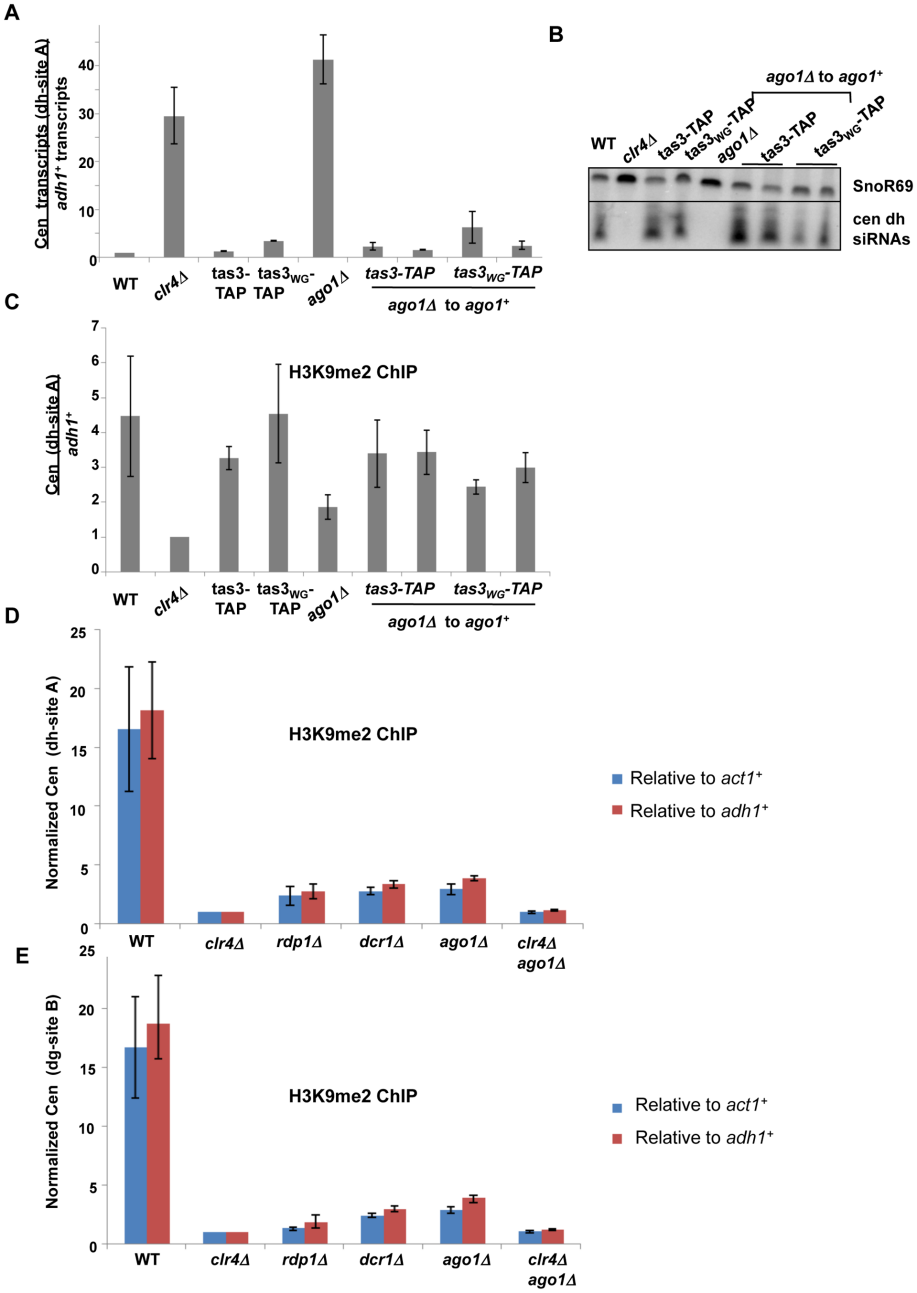
Ago1 reintegration into *tas3_WG_* cells promotes heterochromatin assembly, and *ago1* null cells retain significant centromeric H3K9me2. A. Real time PCR analysis of cDNA derived from the indicated strains, measuring centromeric *dh* transcripts normalized to *adh1^+^*, and to a wild type strain (PY 42). Data represents the mean ± SEM from analysis of 2 independent cDNA preparations from duplicate biological samples. Strains used: PY 42, 1798, 1064, 2267, 901, 5193, 5194, 5202, 5203. See also Figure S5A. B. Small RNA Northern blot to identify centromeric *dh* siRNAs, with SnoR69 as a loading control. Samples used as in (A). C. ChIP for H3K9me2 on centromeric *dh* repeats, assessed by real time PCR and normalized to *adh1* association. Data represents mean of duplicate independent ChIP experiments ± SEM. Strains as listed in (A). D. ChIP for H3K9me2 on centromeric *dh* repeats, assessed by real time, and normalized to both *act1^+^* and *adh1^+^* association. Data represent mean of 4 independent ChIP experiments ± SEM. Strains analyzed are PY42, 1798, 1478, 1550, 901, 5236. Two tailed *P* value for *ago1Δ* vs *clr4Δ* and for *ago1Δ* vs *ago1Δclr4Δ* = <0.0001 in t test for data normalized to *adh1^+^*, and *p* = 0.0053 (*ago1Δ* vs *clr4Δ*) and *p* = 0.0058 (*ago1Δ* vs *ago1Δclr4Δ*) for data normalized to *act1^+^*. E. ChIP for H3K9me2 on centromeric *dg* repeats, as described for (D). Two tailed *P* value for *ago1Δ* vs *clr4Δ* = 0.0005 and for *ago1Δ* vs *ago1Δclr4Δ* = <0.0008 in t test for *adh1* normalized data, and *p* = 0.001 (*ago1Δ* vs *clr4Δ*) and *p* = 0.0016 (*ago1Δ*vs *ago1Δclr4Δ*) for *act1* normalized data.

To test the role of Ago1 in heterochromatin assembly, we first performed transient depletion experiments for Ago1 in the *tas3_WG_-TAP* background (Figure 7). We integrated a genomic clone of *ago1^+^* into *ago1* null *tas3-TAP* and *tas3_WG_-TAP* cells, and monitored heterochromatin assembly. In contrast to *ago1* null cells, where centromeric transcripts are highly elevated (above the levels seen in *clr4Δ* cells), reintroduction of *ago1^+^* into either *tas3-TAP* or *tas3_WG_-TAP* cells reduced centromeric *dh* and *dg* transcripts to levels seen normally in *tas3-TAP* and *tas3_WG_-TAP* cells (Figure 7A and Figure S5A). Consistent with this suppression, we found that unlike *ago1* null cells, where siRNA production is severely reduced, centromeric siRNAs are synthesized at normal levels following reintroduction of *ago1* ^+^ (Figure 7B). Next, we performed ChIP experiments to monitor H3K9me2 levels in *ago1Δ* cells. *ago1* deletion reduces H3K9me2 accumulation at centromeres below that of wild type cells, but above that of *clr4Δ* cells. On reintroduction of *ago1^+^*, centromeric H3K9me2 levels accumulate to normal levels (Figure 7C), similar to the results seen on reintegration of other RNAi components into *tas3_WG_* cells. These data suggest that cells lacking *ago1* behave similarly to other RNAi defective strains, and functionally that there is sufficient H3K9me2 in *ago1Δ tas3_WG_* cells to drive heterochromatin assembly following reintroduction of *ago1^+^*. We further analyzed centromeric H3K9me2 levels in RNAi defective strains. At the 2 sites tested within centromeric *dg* and *dh* repeats, H3K9me2 levels were significantly elevated in *ago1Δ* cells above the background levels in *clr4Δ* or *ago1Δ clr4Δ* cells, and H3K9me2 accumulation at centromeres was similar in all RNAi deficient backgrounds tested. This data would suggest that Ago1, like other RNAi components, is not acting upstream of Clr-C for heterochromatin assembly.

### RNAi pathway is not required for initial step in heterochromatin initiation

**Figure 8:**
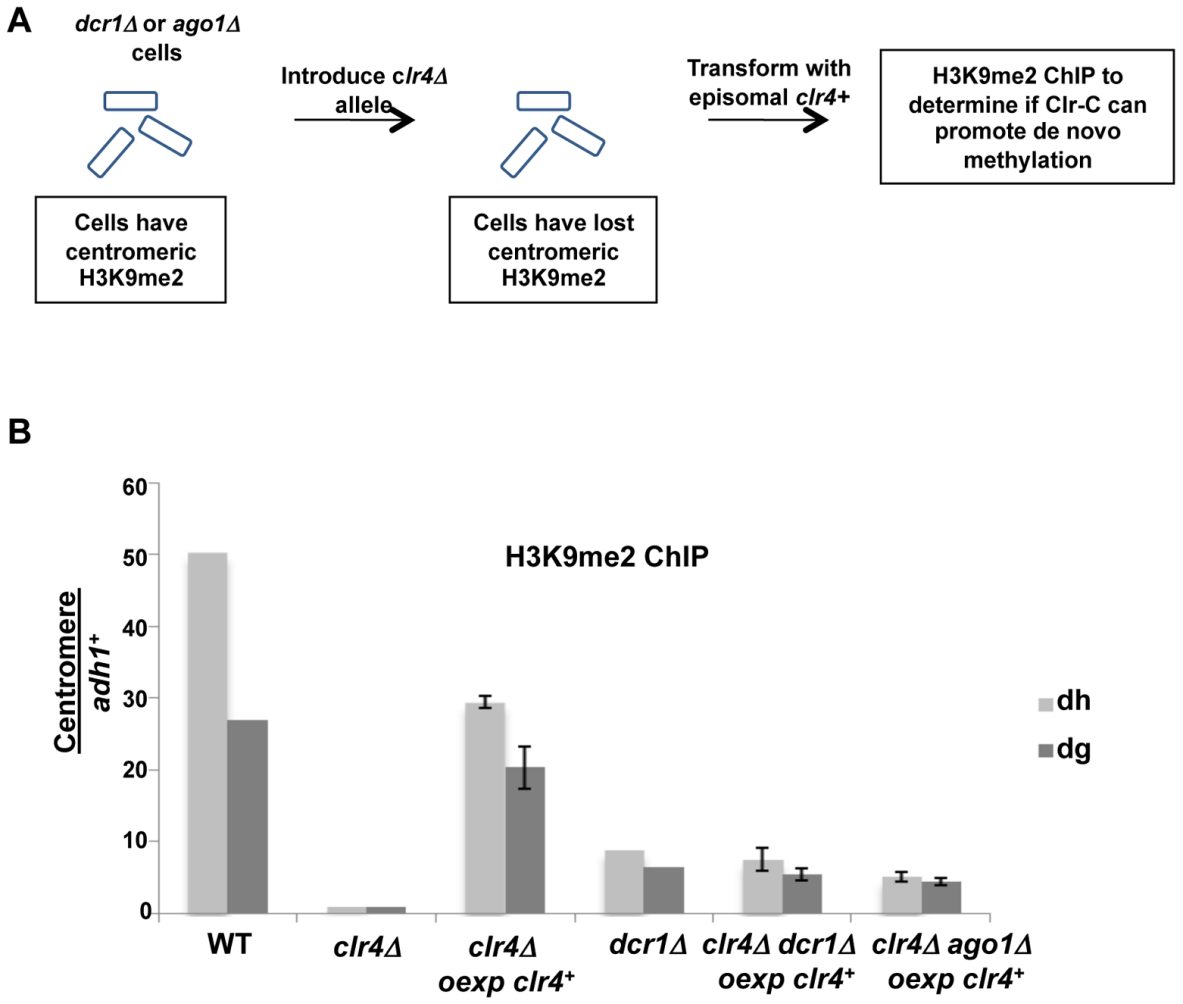
Initial Clr-C recruitment to centromeres can occur independently of *ago1^+^* and *dcr1^+^*. A. Schematic for removal of residual H3K9me2 in *dcr1Δ* or *ago1Δ* cells by introduction of *clr4Δ*, prior to re-expression of genomic *clr4^+^* from episomal vector to test for Clr-C's ability to promote de novo heterochromatin assembly in RNAi-deficient *tas3_WG_* cells. B. Removal of residual H3K9me2 in *dcr1Δ* and *ago1Δ* cells was performed by generation of double mutants with *clr4Δ*, and then ability of Clr-C to generate de novo centromeric H3K9me2 methylation was tested in absence of RNAi pathway following transformation with genomic *clr4^+^* plasmid. H3K9me2 ChIP was performed on indicated strains, and assessed by real time PCR analysis. Strains analyzed: PY12, 5516, 5557, 5517, 1637, 5522, 5523, 5520, 5521. All were grown in PMG-his media. Data represent mean of 3 or 4 ChIPs ±SEM for all samples except *dcr1Δ* and WT for which 1 ChIP is shown.

**Figure 9:**
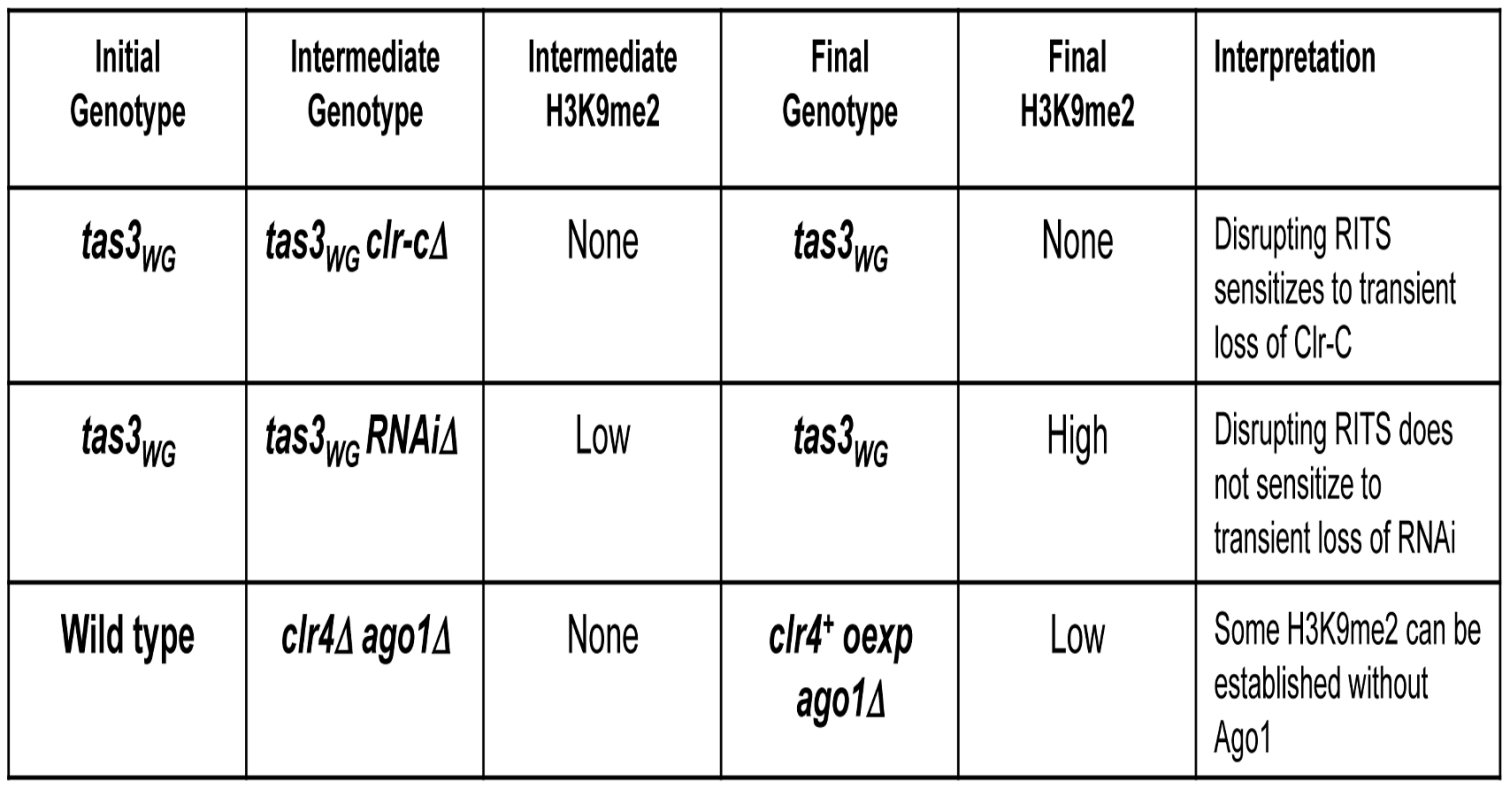
Table summarizing results from this study. Tas3_WG_ cells lacking Clr-C components lack H3K9me, but retain heterochromatin independent siRNAs and priRNAs. These cells cannot support recruitment of Clr-C to centromeres following reintegration of the missing Clr-C component. In contrast, Tas3_WG_ cells lacking RNAi components retain residual H3K9me, but lack siRNAs. On reintegration of the missing RNAi component, these cells convert to allow full heterochromatin assembly on centromeric repeats. These results suggest that Clr-C can function independently of the RNAi pathway in the assembly of centromeric heterochromatin. To further test this hypothesis, H3K9me was withdrawn from RNAi deficient cells to determine whether Clr-C can target centromeres de novo independently of the RNAi pathway. Overexpression of Clr4 in *clr4Δago1Δ* cells supports recruitment of Clr-C activity to centromeric repeats to allow initiation of heterochromatin. These data suggest that normally RNAi-independent and RNAi-dependent mechanisms cooperate for full heterochromatin assembly.

Our demonstration that heterochromatin can assemble following transient depletion of RNAi components in *tas3_WG_* cells is suggestive that the RNAi pathway is acting downstream of Clr-C. However, given that low levels of centromeric H3K9me2 are maintained in RNAi-defective cells, it is difficult to assess whether RNAi is required for the initial step in heterochromatin initiation. To address this question, we removed residual H3K9me2 from RNAi defective cells by introduction of the *clr4Δ* allele. We then tested whether H3K9me2 could be deposited at centromeres following expression of Clr4 in these cells that lack both Clr4 and Ago1 or Clr4 and Dcr1 (Figure 8A). Following overexpression of *clr4* ^+^ in *ago1Δclr4Δ* cells, H3K9me2 could be detected on centromeric repeats above the background observed in *clr4* null cells, and similar to levels found normally in *ago1Δ* cells. Similar results were obtained following overexpression of *clr4* ^+^ in *dcr1Δclr4Δ* cells (Figure 8B). Thus, when overexpressed, Clr4 can target centromeric repeats to initiate H3K9me2 deposition in the absence of a functional RNAi pathway. We note, however, that reintroduction of *clr4^+^* into its normal locus in these cells is not sufficient, in the absence of the RNAi pathway, for accumulation of detectable centromeric H3K9me2 (data not shown). Together, these experiments strongly indicate that Clr-C can initiate H3K9me deposition at centromeres via RNAi-independent mechanisms, but that cooperation between RNAi-dependent and RNAi-independent factors normally results in full heterochromatin assembly (summarized in Figure 9).

## Discussion

We have utilized our novel mutant, *tas3_WG_*, to identify genetic requirements for heterochromatin initiation as opposed to those required for the maintenance of pre-existing heterochromatin. We used an approach in which genes required for heterochromatin formation are deleted and reintroduced and then heterochromatin assembly is examined. In wild-type cells, heterochromatin can be established regardless of the factor removed, indicating that establishment mechanisms are robust to perturbations of the system. However, in cells harboring a disrupted RITS complex, we found that the establishment of silencing becomes sensitive to the prior presence of particular silencing factors. Our data demonstrate that the ability to assemble heterochromatin in such gene removal-restoration experiments in *tas3_WG_* cells correlates with the prior presence of H3K9me2 on centromeric repeats, but does not require the prior presence of small RNA species. These data strongly suggest that RNAi-independent mechanisms of recruitment of Clr-C play a key role in the assembly of centromeric heterochromatin.

We also tested whether RNAi is required in an obligate manner to initiate de novo heterochromatin assembly in cells that lack any prior H3K9me. To accomplish this, we generated *clr4Δ dcr1Δ* cells and found that some deposition of H3K9me2 at centromeric sequences occurred upon overexpression of *clr4^+^* (Figure 8C). These experiments clearly demonstrate that Clr-C can function to initiate de novo centromeric heterochromatin assembly independently of the RNAi pathway.

Recently, Ago1 and its associated priRNAs have been proposed to trigger heterochromatin formation. This notion is partly based on an observation that an *ago1Δ* strain had little or no H3K9me2 at centromeres, suggesting an upstream role for Ago1 [20]. If this hypothesis were correct, we anticipated that in our *tas3_WG_* system, that transient depletion of Ago1 might block heterochromatin assembly similar to what we observed for Clr4. In contrast, we found that *tas3_WG_* cells formed robust centromeric heterochromatin on reintroduction of *ago1^+^* into *ago1Δ* cells (Figure 7A–7C). Consequently, we re-examined the reported critical Dicer-independent role for Ago1 in driving H3K9Me. In contrast to a recent study [20], we found that H3K9me2 levels in *ago1Δ* cells were no lower than in other RNAi-defective backgrounds (Figure 7D and 7E). To further probe for a potential role of priRNAs in heterochromatin initiation, we generated cells that lack both Ago1 (which binds priRNAs) and Clr4, and tested whether reintroduction of Clr4 could promote de novo centromeric H3K9me2 in the absence of Ago1-priRNA targeting activities. This experiment revealed that indeed Clr4, when overexpressed, can initiate H3K9me2 deposition at centromeres independent of Ago1 (Figure 8C).

Taken together, our data demonstrate (1) that Clr-C components can act independently of members of the RNAi pathway to initiate heterochromatin assembly and (2) the ability to promote heterochromatin assembly in *tas3_WG_* cells correlates with the prior levels of centromeric H3K9me2 in mutant backgrounds, and not the initial small RNA abundance and (3), that Ago1-bound priRNAs are unlikely to be the key initiator of heterochromatin assembly. The functional data that we present therefore counters the model for heterochromatin initiation proposed recently [20], and supports that RNAi-independent factors, together with the RNAi pathway, are necessary for full heterochromatin assembly.

The conclusions that derived from our observations contrast with the widely held belief that RNAi initiates heterochromatin assembly at fission yeast centromeres. Although it has been shown by several labs that small RNAs derived from exogenous hairpin RNAs can induce silencing of genomic loci [37], [38], these effects tend to be very weak and very locus specific. In these experiments, silencing efficiency correlates with proximity to sites of heterochromatin, or is enhanced by overexpression of heterochromatin proteins.

The production of the majority of centromeric small RNAs depends on the presence of heterochromatin. However, low levels of small RNAs are found in Clr-C deletion backgrounds [12], [20] or in histone H3K9R mutant cells [19]. Interestingly, Clr-C mutants that completely lack H3K9me are deficient for heterochromatin establishment in our reintegration assay, in spite of the presence of centromeric siRNAs (which are below the level of detection of our Northern assay). In contrast, RNAi-defective strains that are devoid of, or express even lower levels of centromeric small RNAs than Clr-C mutants [12], [19], [20], can assemble heterochromatin effectively following reintegration of the wild type gene into the *tas3_WG_* background.

Although not easy to detect, priRNAs, which have been postulated to prime heterochromatin establishment, are expected to be present in all of the genetic backgrounds that we tested for heterochromatin initiation. This class of small RNAs therefore does not appear to contribute to the differential ability of mutants to initiate heterochromatin assembly in the *tas3_WG_* background. Finally, our data showing that transient depletion of *ago1* ^+^ does not impair heterochromatin establishment in *tas3_WG_* cells, and that *ago1Δ* cells retain H3K9me2, would argue that *ago1* ^+^ and priRNAs are not the initiating trigger for heterochromatin assembly.

These results beg the question of how low levels of siRNA synthesis occur in the absence of heterochromatin, since early models suggested that localization of the RITS and RDRC complexes to centromeres was a prerequisite for centromeric siRNA generation, and that RITS and RDRC complex localization was dependent on Clr4 [8], [11]. One study suggested that single-stranded transcripts from centromeric sequences can adopt secondary structures to yield dsRNA that can be targeted by Dcr1 to form siRNAs [19]. Such models for the heterochromatin-independent synthesis of centromeric siRNAs may now help to explain our previously puzzling result that Chp1 chromodomain mutants that are defective for heterochromatin establishment following transient depletion of *clr4^+^* express abundant siRNAs [6].

How low levels of H3K9me2 are initially placed at centromeres remains an open question, but our data supports that it is not absolutely dependent on small RNAs and the RNAi pathway. We suggest that H3K9me2 deposition is linked to the transcription of the centromere. Mutants in three separate components of the RNA pol II complex show defects in heterochromatin assembly, including a mutant that truncates the C terminal repetitive tail of the largest subunit of the polymerase, Rpb1 [16]–[18]. This raises the intriguing possibility that, similar to other histone modifying enzymes such as the Set1 and Set2 methyltransferases, that Clr-C may associate with, and be brought to chromatin, via RNA polymerase II [39]. Another mutation in RNA pol II that causes defective heterochromatin assembly resides in the Rpb7 subunit [16], which together with its partner, Rpb4, is thought to be an accessory and non-obligate component of yeast RNA pol II. One interesting possibility would be if Clr-C recruitment to regions of chromatin that are destined to become heterochromatic was controlled by modulation of RNA pol II by the Rpb4/7 subcomplex. In contrast, in plants, two pol II-related RNA polymerase activities, Pol IV and Pol V, have evolved to mediate heterochromatin assembly on repetitive sequences (reviewed in [40]).

## Materials and Methods

### Plasmid construction

Integration plasmids for genomic clones were constructed by PCR using Phusion polymerase (NEB) and standard cloning or Gateway (Invitrogen) techniques. Oligonucleotide sequences are listed in Table S2. Full details of plasmid construction are listed in Text S1.

### Strain generation

Strains used in this study are listed in Table S1 and details of their construction and verification are in Text S1.

### Serial dilution assays on selective media

Cells were cultured overnight at 25°C in rich YES medium to a density of approximately 5×10^6^ cells/ml. Cells were washed extensively in PMG media, counted, and five-fold serial dilutions made, such that plating of 4 ul of cells yielded 1.2×10**^4^** cells within the most concentrated spot. Plating was performed on PMG complete media, PMG media lacking uracil, and PMG complete media supplemented with 2g FOA per liter as described previously [21], and incubated for 5 days at 25°C.

### Transcript and siRNA analyses

Transcript and siRNA analyses were performed as previously described [10], [21]. Oligos for real time PCR analysis: (*dh*) JPO-769 and JPO-770, (*dg*) JPO-986, JPO-987, *adh1*, JPO-793 and JPO-794 [10]. RNA was prepared from duplicate cultures for every experiment, and for analyses following gene reintegration, multiple independent re-integrants were assessed.

Real-time PCR was performed on an Eppendorf Mastercycler ep Realplex machine using Quantifast Sybr green (Qiagen). Data was analyzed using the ΔCt method, ensuring that all samples gave Ct values within the experimentally determined linear range.

### Chromatin Immunoprecipitation analyses

Chromatin immunoprecipitation was performed as previously described [10], [21], using antibodies that recognize H3K9me2 (Abcam) and Chp1 (Abcam). Further details are in Text S1.

## Supporting Information

Figure S1 Analysis of transcript accumulation from *dg* sites within the centromere by real time PCR analysis. A. Transcripts from cen dg sequences were measured by real time PCR relative to *adh1*+ transcript accumulation in cDNA derived from strains listed in Figure 2C (*raf1Δ* to *raf1* ^+^). Duplicate RNA preparations were used to generate duplicate cDNAs and data represent mean ± SEM. B. Centromeric transcripts from the dg region of the centromere were measured by real time PCR of cDNA derived from strains listed in Figure 2E (*raf2Δ* to *raf2* ^+^). Analysis was performed as described above.
(0.01 MB PDF)

Figure S2 Analysis of strains bearing genomic reintegration of *dcr1* ^+^. A. Serial dilution assay to monitor growth of *cen::ura4* ^+^ reporter strains on non-selective media (complete), media lacking uracil (−URA), or media supplemented with FOA (+FOA). Following reintegration of *dcr1* ^+^ into the genomic *dcr1Δ* locus, both *tas3-TAP* and *tas3_WG_-TAP* isolates were able to grow on FOA. Strains used were PY2036, 3310, 3307, 3501, 3502, 3499, 3500. B. Real time PCR analysis of centromeric transcript accumulation from the *dh* repeat sequences relative to *adh1* ^+^ transcript accumulation in cDNA derived from the indicated strains. Two independent reintegrants of *dcr1* ^+^ (*dcr1Δ* to *dcr1* ^+^) were analyzed for both the *tas3-TAP* and *tas3^WG^-TAP* backgrounds. Data represent mean ± SEM for cDNA samples from 2 independent RNA preparations for each strain. Data was normalized to wild type *cen::ura4^+^* strain (PY2036), which was set at 1. Strains used were PY2036, 3310, 3307, 3501, 3502, 3499, 3500. C. Centromeric *dg* transcripts were measured by similar methods and using the same cDNA samples as used for (B). D. Northern blotting for small RNA species in RNA preparations from the strains listed in (B). Blot was probed for siRNAs derived from *dh* repeats and for the snoR69 RNA as a loading control.
(0.24 MB PDF)

Figure S3 Analysis of centromeric *dg* and *dh* transcript accumulation by real time PCR. A. Real time PCR analysis of *dg* centromeric transcripts relative to *adh1* ^+^ in cDNA derived from strains listed in Figure 5C (*cid12Δ* to *cid12* ^+^). Duplicate RNA preparations were used to generate duplicate cDNAs and data represent mean ± SEM. B. Real time PCR analysis of transcripts derived from dg centromeric repeats relative to *adh1* ^+^ in strains listed in Figure 5E (*hrr1Δ* to *hrr1* ^+^). Analysis was performed as described above.
(0.02 MB PDF)

Figure S4 Analysis of centromeric transcript accumulation in *rdp1* null cells and Chp1 association with centromeric *dh* sequences in *hrr1* mutant cells. A. Real time PCR analysis of transcripts derived from *dh* centromeric repeats relative to *adh1* ^+^ in strains listed in Figure 6C *(rdp1Δ* to *rdp1* ^+^). Analysis was performed as described in Figure 6C. B. ChIP was performed with Chp1 antibodies on chromatin prepared from strains listed in Figure 6D. Real time PCR was used to quantify Chp1 association with *dh* relative to the euchromatic *adh1* control. Data was normalized to cells lacking *clr4* (set at 1), in which Chp1 does not associate with centromeres. Error bars represent the SEM of duplicate ChIPs using different biological samples.
(0.02 MB PDF)

Figure S5 Transient depletion of *ago1* ^+^ does not affect establishment of silencing of centromeric transcripts in *tas3_WG_* cells. A. Real Time PCR analysis of cDNA prepared from indicated strains, measuring centromeric *dg* transcript accumulation normalized to *adh1* ^+^ expression. Data represents mean average of analysis of 2 independent cDNA preparations from duplicate biological samples, measuring two independent *ago1* ^+^ reintegrants for each background, with error bars representing SEM. Strains used were as in Figure 7A.
(0.01 MB PDF)

Table S1 Strains used in this study.
(0.11 MB DOC)

Table S2 Oligonucleotide sequences.
(0.06 MB DOC)

Text S1 Supplemental experimental procedures.
(0.05 MB DOC)
