# Supplementary figures and images for: Continuous Requirement for the Clr4 Complex But Not RNAi for Centromeric Heterochromatin Assembly in Fission Yeast Harboring a Disrupted RITS Complex
Source: PLoS Genet. 2010 Oct 28;6(10):e1001174. doi: 10.1371/journal.pgen.1001174 (PMC2965749; doi:10.1371/journal.pgen.1001174)

Figure S1

A

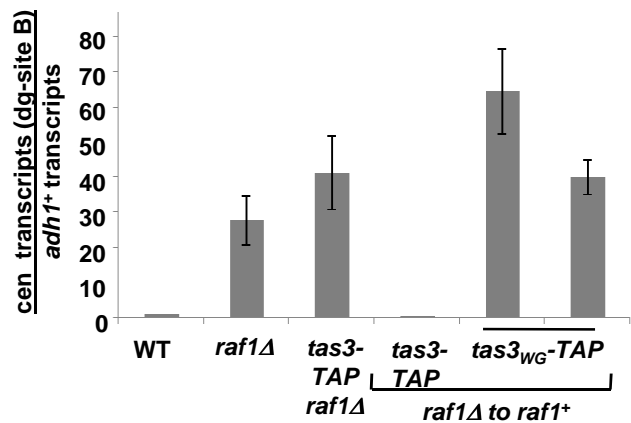

B

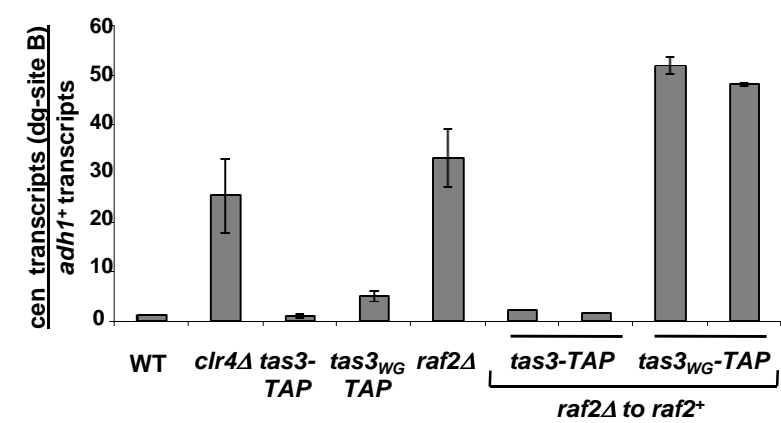

Supplement: Figure S1 — Analysis of transcript accumulation from dg sites within the centromere by real time PCR analysis. A. Transcripts from cen dg sequences were measured by real time PCR relative to adh1+ transcript accumulation in cDNA derived from strains listed in Figure 2C (raf1Δ to raf1 +). Duplicate RNA preparations were used to generate duplicate cDNAs and data represent mean ± SEM. B. Centromeric transcripts from the dg region of the centromere were measured by real time PCR of cDNA derived from strains listed in Figure 2E (raf2Δ to raf2 +). Analysis was performed as described above. (0.01 MB PDF) [file pgen.1001174.s001.pdf]

Figure S2

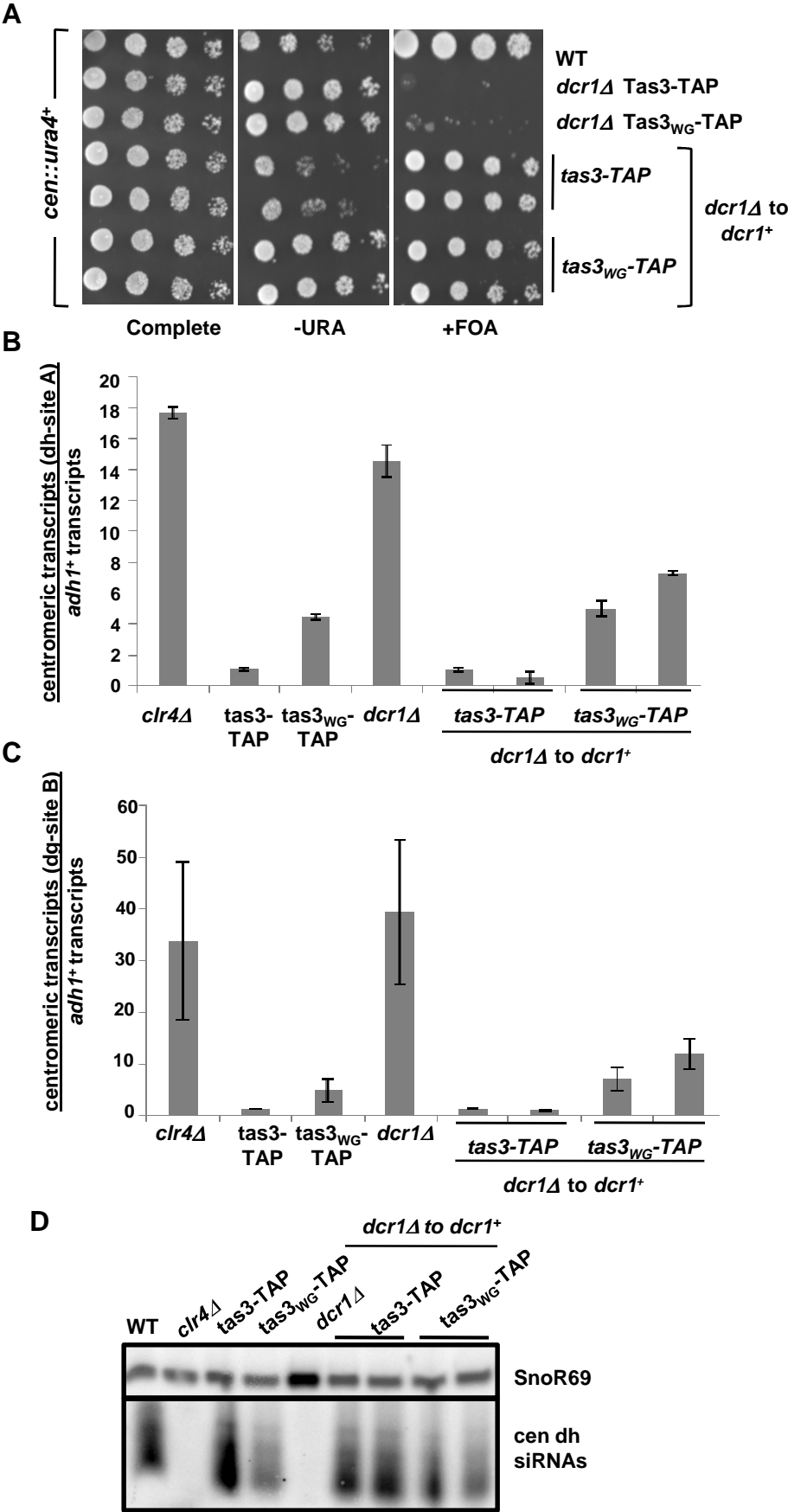

Supplement: Figure S2 — Analysis of strains bearing genomic reintegration of dcr1 +. A. Serial dilution assay to monitor growth of cen::ura4 + reporter strains on non-selective media (complete), media lacking uracil (−URA), or media supplemented with FOA (+FOA). Following reintegration of dcr1 + into the genomic dcr1Δ locus, both tas3-TAP and tas3WG-TAP isolates were able to grow on FOA. Strains used were PY2036, 3310, 3307, 3501, 3502, 3499, 3500. B. Real time PCR analysis of centromeric transcript accumulation from the dh repeat sequences relative to adh1 + transcript accumulation in cDNA derived from the indicated strains. Two independent reintegrants of dcr1 + (dcr1Δ to dcr1 +) were analyzed for both the tas3-TAP and tas3WG-TAP backgrounds. Data represent mean ± SEM for cDNA samples from 2 independent RNA preparations for each strain. Data was normalized to wild type cen::ura4+ strain (PY2036), which was set at 1. Strains used were PY2036, 3310, 3307, 3501, 3502, 3499, 3500. C. Centromeric dg transcripts were measured by similar methods and using the same cDNA samples as used for (B). D. Northern blotting for small RNA species in RNA preparations from the strains listed in (B). Blot was probed for siRNAs derived from dh repeats and for the snoR69 RNA as a loading control. (0.24 MB PDF) [file pgen.1001174.s002.pdf]

Figure S3

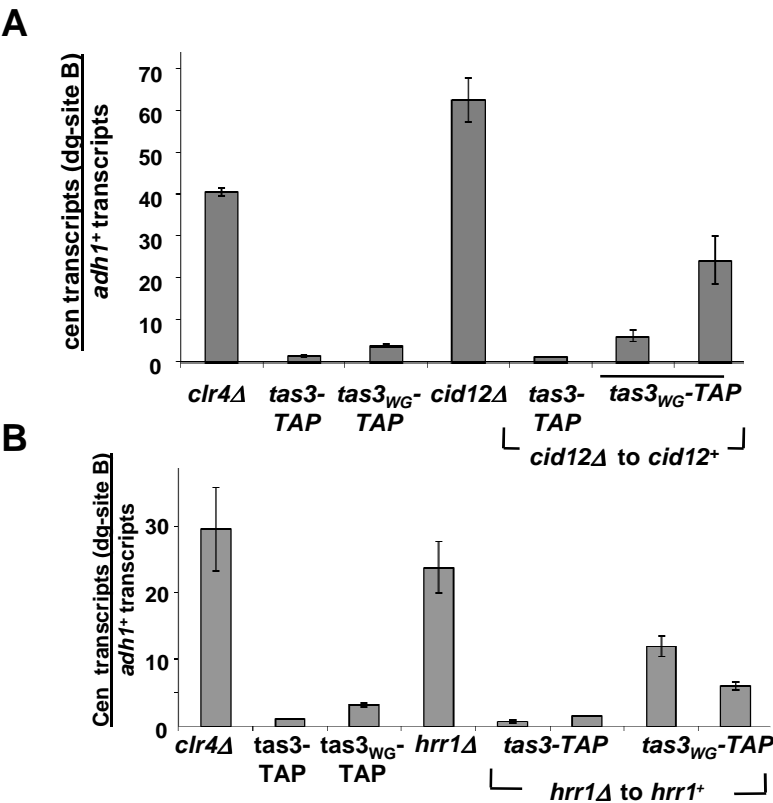

Supplement: Figure S3 — Analysis of centromeric dg and dh transcript accumulation by real time PCR. A. Real time PCR analysis of dg centromeric transcripts relative to adh1 + in cDNA derived from strains listed in Figure 5C (cid12Δ to cid12 +). Duplicate RNA preparations were used to generate duplicate cDNAs and data represent mean ± SEM. B. Real time PCR analysis of transcripts derived from dg centromeric repeats relative to adh1 + in strains listed in Figure 5E (hrr1Δ to hrr1 +). Analysis was performed as described above. (0.02 MB PDF) [file pgen.1001174.s003.pdf]

Figure S4

A

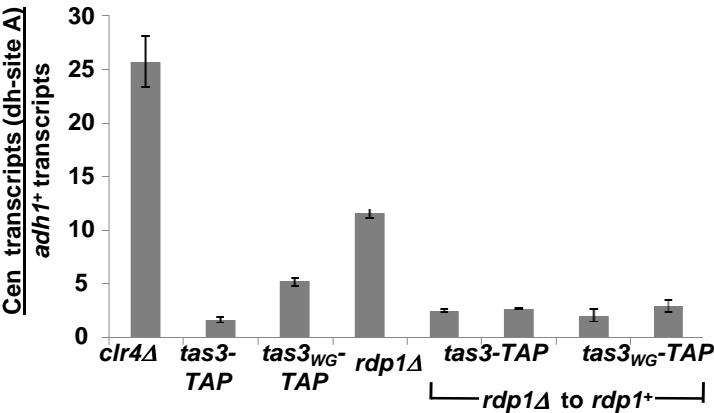

B

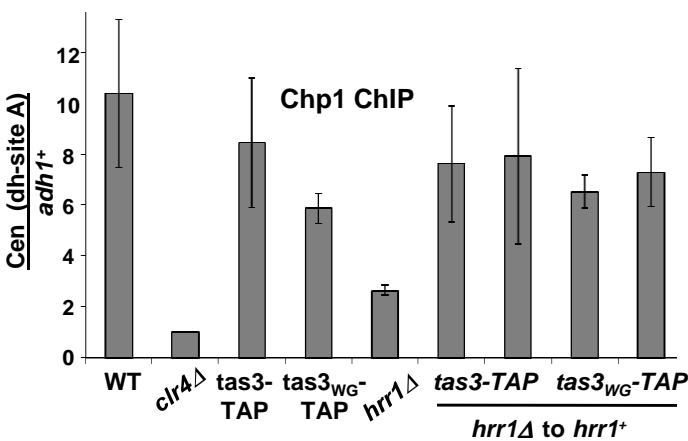

Supplement: Figure S4 — Analysis of centromeric transcript accumulation in rdp1 null cells and Chp1 association with centromeric dh sequences in hrr1 mutant cells. A. Real time PCR analysis of transcripts derived from dh centromeric repeats relative to adh1 + in strains listed in Figure 6C (rdp1Δ to rdp1 +). Analysis was performed as described in Figure 6C. B. ChIP was performed with Chp1 antibodies on chromatin prepared from strains listed in Figure 6D. Real time PCR was used to quantify Chp1 association with dh relative to the euchromatic adh1 control. Data was normalized to cells lacking clr4 (set at 1), in which Chp1 does not associate with centromeres. Error bars represent the SEM of duplicate ChIPs using different biological samples. (0.02 MB PDF) [file pgen.1001174.s004.pdf]

Figure S5

A

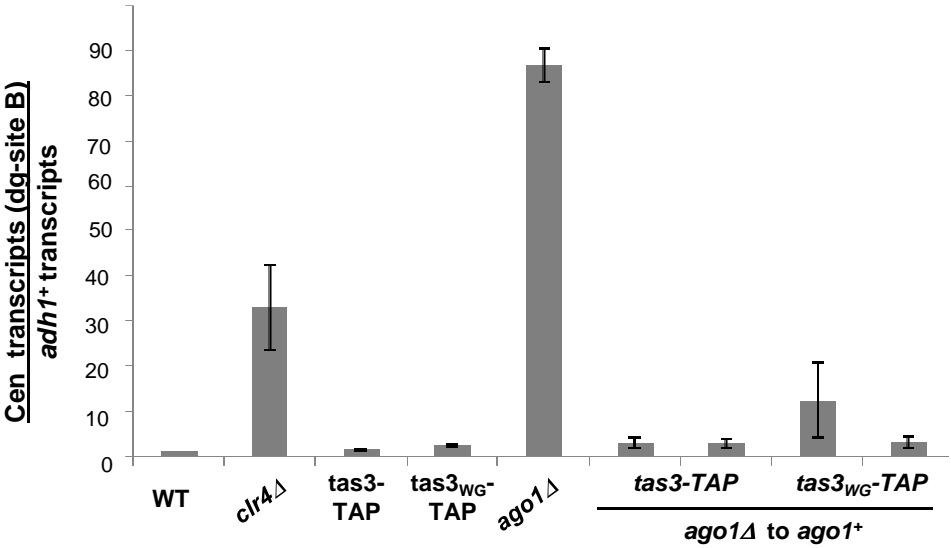

Supplement: Figure S5 — Transient depletion of ago1 + does not affect establishment of silencing of centromeric transcripts in tas3WG cells. A. Real Time PCR analysis of cDNA prepared from indicated strains, measuring centromeric dg transcript accumulation normalized to adh1 + expression. Data represents mean average of analysis of 2 independent cDNA preparations from duplicate biological samples, measuring two independent ago1 + reintegrants for each background, with error bars representing SEM. Strains used were as in Figure 7A. (0.01 MB PDF) [file pgen.1001174.s005.pdf]
