# Supplementary material for: Continuous Requirement for the Clr4 Complex But Not RNAi for Centromeric Heterochromatin Assembly in Fission Yeast Harboring a Disrupted RITS Complex
Source: PLoS Genet. 2010 Oct 28;6(10):e1001174. doi: 10.1371/journal.pgen.1001174 (PMC2965749; doi:10.1371/journal.pgen.1001174)
Supplement: Table S1 — Strains used in this study. (0.11 MB DOC) [file pgen.1001174.s006.doc]

**Table S1 : Strains used in this study**

| PY 2036 | h- otr1R Sph1::ura4 ura4-DS/E ade6-210 leu1-32 his3+ |
| --- | --- |
| PY 3776 | h- otr1R Sph1::ura4 ura4-D18 tas3 tap-KanR rik1::LEU2 ade6-210 leu1-32 his3Δ |
| PY 3778 | h- otr1R Sph1::ura4 ura4-D18 tas3 W265A,G266A-tap- KanR rik1::LEU2 ade6-210 leu1-32 his3Δ |
| PY 4879 | h- otr1R Sph1::ura4 ura4-D18 tas3 tap-KanR JP1287 his3+ genomic rik1+(Pst1)::rik1::LEU2 ade6? leu1-32 his3Δ |
| PY 4880 | h- otr1R Sph1::ura4 ura4-D18 tas3 tap-KanR JP1287 his3+ genomic rik1+(Pst1)::rik1::LEU2 ade6? leu1-32 his3Δ |
| PY 4881 | h- otr1R Sph1::ura4 ura4-D18 tas3 W265A,G266A-tap-KanR JP1287 his3+ genomic rik1+(Pst1):: rik1::LEU2 ade6? leu1-32 his3Δ |
| PY 4882 | h- otr1R Sph1::ura4 ura4-D18 tas3 W265A,G266A-tap-KanR JP1287 his3+ genomic rik1+(Pst1):: rik1::LEU2 ade6? leu1-32 his3Δ |
| PY 1838 | h- otr1R Sph1::ura4 ura4-DS/E clr4Δ::KanR ade6-210 leu1-32 his3Δ |
| PY 3494 | h- otr1R Sph1::ura4 ura4-DS/E tas3-tap-KanR ade6-210 leu1-32 his3Δ |
| PY 3497 | h- otr1R Sph1::ura4 ura4-DS/E tas3 W265A,G266A-tap-KanR ade6-210 leu1-32 his3Δ  |
| PY 3287 | h+ otr1R Sph1::ura4 ura4-DS/E raf1Δ::KanR ade6-210 leu1-32 |
| PY 3659 | h- otr1R Sph1::ura4 ura4-DS/E tas3-tap-KanR raf1Δ::KanR ade6-210 leu1-32 his3Δ |
| PY 3707 | h- otr1R Sph1::ura4 ura4-DS/E tas3-tap-KanR JP1172 his3+ genomic raf1+(PstI)::raf1Δ::KanR ade6-210 leu1-32 his3Δ |
| PY 3708 | h- otr1R Sph1::ura4 ura4-DS/E tas3-tap-KanR JP1172 his3+ genomic raf1+(PstI)::raf1Δ::KanR ade6-210 leu1-32 his3Δ  |
| PY 3710 | h- otr1R Sph1::ura4 ura4-DS/E tas3 W265A,G266A-tap-KanR JP1172 his3+ genomic raf1+(PstI)::raf1Δ::KanR ade6-210 leu1-32 his3Δ |
| PY 3711 | h- otr1R Sph1::ura4 ura4-DS/E tas3 W265A,G266A-tap-KanR JP1172 his3+ genomic raf1+(PstI)::raf1Δ::KanR ade6-210 leu1-32 his3Δ |
| PY 3675 | h- otr1R Sph1::ura4 ura4-DS/E tas3-tap-KanR raf2Δ::KanR ade6-210 leu1-32 his3Δ |
| PY 3676 | h- otr1R Sph1::ura4 ura4-DS/E tas3 W265A,G266A-tap-KanR raf2Δ::KanR ade6-210 leu1-32 his3Δ |
| PY 3781 | h- otr1R Sph1::ura4 ura4-DS/E tas3-tap-KanR JP1178 his3+ genomic raf2+(EagI)::raf2Δ::KanR ade6-210 leu1-32 his3Δ |
| PY 3783 | h- otr1R Sph1::ura4 ura4-DS/E tas3-tap-KanR JP1178 his3+ genomic raf2+(EagI)::raf2Δ::KanR ade6-210 leu1-32 his3Δ |
| PY 3791 | h- otr1R Sph1::ura4 ura4-DS/E tas3 W265A,G266A-tap-KanR JP1178 his3+ genomic raf2+(EagI)::raf2Δ::KanR ade6-210 leu1-32 his3Δ |
| PY 3792 | h- otr1R Sph1::ura4 ura4-DS/E tas3 W265A,G266A-tap-KanR JP1178 his3+ genomic raf2+(EagI)::raf2Δ::KanR ade6-210 leu1-32 his3Δ |
| PY 3293 | h- otr1R Sph1::ura4 ura4-DS/E raf2Δ::KanR ade6-210 leu1-32 |
| PY 41 | h+ ura4-D18 ade6-210 leu1-32 arg3Δ his3Δ |
| PY 1065 | h+ tas3-TAP-KanR ura4-DS/E ade6-210 leu1-32 arg3Δ his3Δ |
| PY 2268 | h+ tas3 W265A,G266A-tap-KanR ura4-D18 ade6-210 leu1-32 arg3Δ his3Δ |
| PY 1797 | h90 clr4Δ::KanR ura4-DS/E ade6-210 leu1-32 arg3Δ his3Δ |
| PY 3515 | h90 pcu4Δ::ura4+ ura4D18 |
| PY 5082 | h90 tas3-tap-KanR JP1173 his3+ genomic pcu4+(PstI)::pcu4Δ::ura4+ ura4DS/E or D18 ade6-210 arg3Δ his3Δ |
| PY 5083 | h90 tas3-tap-KanR JP1173 his3+ genomic pcu4+(PstI)::pcu4Δ::ura4+ ura4DS/E or D18 ade6-210 arg3Δ his3Δ |
| PY 5085 | h90 tas3 W265A,G266A-tap-KanR JP1173 his3+ genomic pcu4+(PstI)::pcu4Δ::ura4+ ura4DS/E or D18 ade6-210 arg3Δ his3Δ |
| PY 5086 | h90 tas3 W265A,G266A-tap-KanR JP1173 his3+ genomic pcu4+(PstI)::pcu4Δ::ura4+ ura4DS/E or D18 ade6-210 arg3Δ his3Δ |
| PY 2678 | h- otr1R Sph1::ura4 ura4-DS/E tas3-tap-KanR JP1084 his3+ genomic clr4 (HpaI)::clr4Δ::KanR ade6-210 leu1-32 his3Δ |
| PY 2679 | h- otr1R Sph1::ura4 ura4-DS/E tas3-tap-KanR JP1084 his3+ genomic clr4 (HpaI)::clr4Δ::KanR ade6-210 leu1-32 his3Δ |
| PY 2680 | h- otr1R Sph1::ura4 ura4-DS/E tas3 W265A,G266A-tap-KanR JP1084 his3+ genomic clr4 (HpaI)::clr4Δ::KanR ade6-210 leu1-32 his3Δ |
| PY 2681 | h- otr1R Sph1::ura4 ura4-DS/E tas3 W265A,G266A-tap-KanR JP1084 his3+ genomic clr4 (HpaI)::clr4Δ::KanR ade6-210 leu1-32 his3Δ |
| PY 4338 | h- otr1R Sph1::ura4 ura4-DS/E tas3 tap-KanR cid12Δ::KanR ade6-210 leu1-32 his3Δ |
| PY 4340 | h- otr1R Sph1::ura4 ura4-DS/E tas3 W265A,G266A-tap-KanR cid12Δ::KanR ade6-210 leu1-32 his3Δ  |
| PY 4423 | h- otr1R Sph1::ura4 ura4-DS/E tas3-tap-KanR JP1257 his3+ genomic cid12+(PstI)::cid12Δ::KanR ade6-210 leu1-32 his3Δ  |
| PY 4424 | h- otr1R Sph1::ura4 ura4-DS/E tas3 W265A,G266A-tap-KanR JP1257 his3+ genomic cid12+(PstI)::cid12Δ::KanR ade6-210 leu1-32 his3Δ |
| PY 4425 | h- otr1R Sph1::ura4 ura4-DS/E tas3 W265A,G266A-tap-KanR JP1257 his3+ genomic cid12+(PstI)::cid12Δ::KanR ade6-210 leu1-32 his3Δ |
| PY 4334 | h- otr1R Sph1::ura4 tas3 tap-KanR hrr1Δ::KanR ade6-210 leu1-32 ura4 DS/E his3Δ |
| PY 4337 | h- otr1R Sph1::ura4 ura4-DS/E tas3 W265A,G266A-tap-KanR hrr1Δ::KanR ade6-210 leu1-32 his3Δ |
| PY 4420 | h- otr1R Sph1::ura4 ura4-DS/E tas3-tap-KanR JP1259 his3+ genomic hrr1+(MluI)::hrr1Δ::KanR ade6-210 leu1-32 his3Δ  |
| PY 4481 | h- otr1R Sph1::ura4 ura4-DS/E tas3-tap-KanR JP1259 his3+ genomic hrr1+(MluI)::hrr1Δ::KanR ade6-210 leu1-32 his3Δ |
| PY 4586 | h- otr1R Sph1::ura4 ura4-DS/E tas3 W265A,G266A-tap-KanR JP1259 his3+ genomic hrr1+(MluI)::hrr1Δ::KanR ade6-210 leu1-32 his3Δ |
| PY 4587 | h- otr1R Sph1::ura4 ura4-DS/E tas3 W265A,G266A-tap-KanR JP1259 his3+ genomic hrr1+(MluI)::hrr1Δ::KanR ade6-210 leu1-32 his3Δ |
| PY 4312 | h- otr1R Sph1::ura4 ura4-DS/E cid12Δ::KanR ade6-210 leu1-32 |
| PY 4308 | h- otr1R Sph1::ura4 ura4-DS/E hrr1Δ::KanR ade6-210 leu1-32 |
| PY 4300 | h- otr1R Sph1::ura4 ura4-DS/E rdp1Δ::KanR tas3-tap-KanR leu1-32 ade6-210 his3Δ  |
| PY 4304 | h- otr1R Sph1::ura4 ura4-DS/E rdp1Δ::kanR tas3 W265A,G266A-tap-KanR leu1-32 ade6-210 his3Δ |
| PY 4401 | h- otr1R Sph1::ura4 ura4-DS/E tas3-tap-KanR JP1250 his3+ genomic rdp1+(NheI)::rdp1Δ::KanR ade6-210 leu1-32 his3Δ |
| PY 4402 | h- otr1R Sph1::ura4 ura4-DS/E tas3-tap-KanR JP1250 his3+ genomic rdp1+(NheI)::rdp1Δ::KanR ade6-210 leu1-32 his3Δ |
| PY 4403 | h- otr1R Sph1::ura4 ura4-DS/E tas3 W265A,G266A-tap-KanR JP1250 his3+ genomic rdp1+(NheI):: rdp1Δ::KanR ade6-210 leu1-32 his3Δ |
| PY 4404 | h- otr1R Sph1::ura4 ura4-DS/E tas3 W265A,G266A-tap-KanR JP1250 his3+ genomic rdp1+(NheI):: rdp1Δ::KanR ade6-210 leu1-32 his3Δ |
| PY 4274 | h- otr1R Sph1::ura4 ura4-DS/E rdp1Δ::KanR ade6-210 leu1-32 his3+ |
| PY 3235 | h- otr1R Sph1::ura4 ura4-DS/E dcr1Δ::KanR ade6-210 leu1-32 his3Δ |
| PY 3501 | h- otr1R Sph1::ura4 ura4-DS/E tas3-tap-KanR JP1162 his3+ genomic dcr1+(NcoI)::dcr1Δ::KanR ade6-210 leu1-32 his3Δ |
| PY 3502 | h- otr1R Sph1::ura4 ura4-DS/E tas3-tap-KanR JP1162 his3+ genomic dcr1+(NcoI)::dcr1Δ::KanR ade6-210 leu1-32 his3Δ |
| PY 3499 | h- otr1R Sph1::ura4 ura4-DS/E tas3 W265A,G266A-tap-KanR JP1162 his3+ genomic dcr1+(NcoI)::dcr1Δ::KanR ade6-210 leu1-32 his3Δ |
| PY 3500 | h- otr1R Sph1::ura4 ura4-DS/E tas3 W265A,G266A-tap-KanR JP1162 his3+ genomic dcr1+(NcoI)::dcr1Δ::KanR ade6-210 leu1-32 his3Δ |
| PY 42 | h- ura4-D18 leu1-32 ade6-210 arg3Δ his3Δ |
| PY 1798 | h- clr4Δ::KanR ura4DS/E leu1-32 ade6-210 arg3Δ his3Δ |
| PY 1064 | h- tas3-TAP-KanR ura4D18 leu1-32 ade6-210 arg3Δ his3Δ |
| PY 2267 | h- tas3 W265A,G266A-tap-KanR ura4D18 leu1-32 ade6-210 arg3Δ his3Δ |
| PY 901 | h- ago1Δ::ura4+ ura4D18 leu1-32 ade6-210 arg3Δ his3Δ |
| PY 5193 | h- tas3 -tap-KanR JP1425 his3+ genomic ago1+(Spe1)::ago1Δ::ura4+ ura4D18 leu1-32 arg3Δ his3Δ |
| PY 5194 | h- tas3 -tap-KanR JP1425 his3+ genomic ago1+(Spe1)::ago1Δ::ura4+ ura4D18 leu1-32 arg3Δ his3Δ |
| PY 5202 | h- tas3 W265A,G266A-tap-KanR JP1425 his3+ genomic ago1+(Spe1)::ago1Δ::ura4+ ura4D18 leu1-32 arg3Δ his3Δ |
| PY 5203 | h- tas3 W265A,G266A-tap-KanR JP1425 his3+ genomic ago1+(Spe1)::ago1Δ::ura4+ ura4D18 leu1-32 arg3Δ his3Δ |
| PY 3310 | h- otr1R Sph1::ura4+ tas3-tap-KanR dcr1Δ::KanR ura4 DS/E ade6-210 leu1-32 his3Δ |
| PY 3307 | h- otr1R Sph1::ura4+ tas3 W265A,G266A-tap-KanR dcr1Δ::KanR ura4 DS/E ade6-210 leu1-32 his3Δ |
| PY 3518 | h- otr1R Sph1::ura4+ tas3 W265A,G266A-tap-KanR raf1Δ::KanR ura4 DS/E ade6-210 leu1-32 his3Δ |
| PY 3776 | h- otr1R Sph1::ura4 tas3 tap-KanR rik1::LEU2 ura4-D18 ade6? his3Δ leu1-32 |
| PY 3778 | h- otr1R Sph1::ura4 tas3 W265A,G266A tap-KanR rik1::LEU2 ura4-D18 ade6? leu1-32 his3Δ |
| PY 5080 | h90 pcu4Δ::ura4+ tas3-TAP-KanR ura4DS/E or D18 ade6-210 his3Δ arg3Δ |
| PY 5081 | h90 pcu4Δ::ura4+ tas3 W265A,G266A-tap-KanR ura4D18 ade6-210 his3Δ |
| PY 2211 | h- ago1Δ::ura4+ tas3-tap-KanR ura4D18 or DS/E ade6-210 leu1-32 arg3Δ his3Δ |
| PY 5186 | h- ago1Δ::ura4+ tas3 W265A,G266A-tap-KanR ura4D18 ade6-210 leu1-32 arg3Δ his3Δ |
| PY 1478 | h- rdp1Δ::KanR ura4D18 ade6-210 leu1-32 |
| PY 1550 | h- dcr1Δ::KanR ura4DS/E ade6-210 leu1-32 his3Δ |
| PY 12 | h- |
| PY 5516 | h- JP1045 (empty vector, his3+) in clr4Δ::KanR ade6-210 leu1-32 ura4DS/E arg3Δ his3Δ  |
| PY 5557 | h- JP1045 (empty vector, his3+) in clr4Δ::KanR ade6-210 leu1-32 ura4DS/E arg3Δ his3Δ  |
| PY 5517 | h- JP1078 (genomic clr4+, his3+) in clr4Δ::KanR ade6-210 leu1-32 ura4DS/E arg3Δ his3Δ |
| PY 1637 | h- dcr1Δ::KanR R.Int::ura4+ ura4DS/E ade6-210 leu1-32 |
| PY 5522 | h- JP1078 (genomic clr4+, his3+) in dcr1Δ::KanR clr4Δ::KanR ura4DS/E leu1-32 ade6-210 his3Δ |
| PY 5523 | h- JP1078 (genomic clr4+, his3+) in dcr1Δ::KanR clr4Δ::KanR ura4DS/E leu1-32 ade6-210 his3Δ |
| PY 5520 | h- JP1078 (genomic clr4+, his3+) in ago1Δ::ura4+ clr4Δ::KanR ade6-210 leu1-32 arg3Δ his3Δ ura4DS/E or D18 |
| PY 5521 | h- JP1078 (genomic clr4+, his3+) in ago1Δ::ura4+ clr4Δ::KanR ade6-210 leu1-32 arg3Δ his3Δ ura4DS/E or D18 |
