# Supplementary material for: Continuous Requirement for the Clr4 Complex But Not RNAi for Centromeric Heterochromatin Assembly in Fission Yeast Harboring a Disrupted RITS Complex
Source: PLoS Genet. 2010 Oct 28;6(10):e1001174. doi: 10.1371/journal.pgen.1001174 (PMC2965749; doi:10.1371/journal.pgen.1001174)
Supplement: Table S2 — Oligonucleotide sequences. (0.06 MB DOC) [file pgen.1001174.s007.doc]

**Table S2 : Oligonucleotide sequences**

| JPO-769 | ccagaccattacaagcactacatacg |
| --- | --- |
| JPO-770 | gaatcttctcttgaataaaaccgcc |
| JPO-987 | gcgatgccaaacaacaatattg |
| JPO-986 | gatactgataatattgagatccacagcac |
| JPO-793 | aacgtcaagttcgaggaagtcc |
| JPO-794 | agagcgtgtaaatcggtgtgg |
| JPO-2000 | aaccctcagctttgggtctt |
| JPO-2001 | tttgcatacgatcggcaata |
| JPO-1464 | aaggaaaaaagcggccgcaattcatagatattgatactataac |
| JPO-1465 | ccaatgcattggttctgcagagatgctatagctggtgttcgc |
| JPO-1550 | tgaatatctgaatgtagctgaag |
| JPO-1551 | aagcgcatttctctaagtactc |
| JPO-1458 | aaggaaaaaagcggccgcaagctactcagtttgtaaatctatttc |
| JPO-1459 | ccaatgcattggttctgcagtcatagccctaattgctatatctgtc |
| JPO-1479 | atacgtcgtaagagaagcattgac |
| JPO-1552 | tcctcacgtagcaaccgatag |
| JPO-1460 | aaggaaaaaagcggccgctataaatgctgtatgagactaggaag |
| JPO-1461 | ttccgcggtcgctatggccgacgtcgacacaatctcagtcgggcctttagcac |
| JPO-1487 | atcaatttagtagatgttagtg |
| JPO-1556 | agctcgaattggttgcacttc |
| JPO-1125 | ggggacaagtttgtacaaaaaagcaggctattttggccgtttcctttttat |
| JPO-1127 | ataatgattatctctgcagttactattattacaaatttatcagtcaaagg |
| JPO-1126 | ataaatttgtaataatagtaactgcagagataatcattatatcttcaat |
| JPO-1128 | ggggaccactttgtacaagaaagctgggtaaccgtgaaagctcatttgttt |
| JPO-1146 | atggcatgactaatagttcac |
| JPO-1596 | gtttgtccacaggagacaagctg |
| JPO-1129 | ggggacaagtttgtacaaaaaagcaggctcattgtgtcagctatgcaatca |
| JPO-1130 | ggggaccactttgtacaagaaagctgggtcgtatggtccgtattttctgga |
| JPO-1595 | atgccgcccgtacgtgctg |
| JPO-1280 | ccagaacaatgctctcatc |
| JPO-1164 | ggggacaagtttgtacaaaaaagcaggctttcccccaatatggctaatgct |
| JPO-1166 | gtgcaagacgccagttactgcaggcactccgcagggagaagaaacgag |
| JPO-1165 | ctccctgcggagtgcctgcagtaactggcgtcttgcactttcattcaa |
| JPO-1172 | ggggaccactttgtacaagaaagctgggtagaaattacactagagtttctcc |
| JPO-1737 | caccacttccgctagaaac |
| JPO-1729 | tccttgtatgcgcaggaacac |
| JPO-1155 | agcaggctgtcgacgcatatgctagatgtggtttgg |
| JPO-1119 | gctgcgactgcaggcaattagctaac |
| JPO-1118 | gttagctaattgcctgcagtcgcagc |
| JPO-1156 | agctgggtactagttaagaggtgtttatgggcagga |
| JPO-1824 | tcaagagctgttatcgttccac |
| JPO-1794 | aattcattttgcgcacttgcag |
| JPO-1056 | gatcgatcgtcgaccgaatcattctagcataaagcattgg |
| JPO-1057 | gatcgatactagtctaattcatgatcaaaactctctccgtg |
| JPO-1597 | tccttccatgatgtctcagcaagg |
| JPO-1598 | cctcctcgatgacacactttgttc |
| JPO-1879 | gatcgatcgagctcgttgttagtttcggtaagctgtttaacgag |
| JPO-1880 | gatcgatcgtcgacgctagatgaatctgaattagacaaataggtaaattg |
| JPO-1886 | tattcgactttactttactatacc |
| JPO-433 | ttagaccaacacatgcttcgcc |
|  |  |
