# Supplementary material for: Continuous Requirement for the Clr4 Complex But Not RNAi for Centromeric Heterochromatin Assembly in Fission Yeast Harboring a Disrupted RITS Complex
Source: PLoS Genet. 2010 Oct 28;6(10):e1001174. doi: 10.1371/journal.pgen.1001174 (PMC2965749; doi:10.1371/journal.pgen.1001174)
Supplement: Text S1 — Supplemental experimental procedures. (0.05 MB DOC) [file pgen.1001174.s008.doc]

**Supplemental Experimental Procedures**

**Plasmid construction information**

All plasmid inserts were fully sequenced prior to linearization within flanking sequences of the gene and integration. All integration plasmids included a *his3*^+^ marker gene allowing initial selection of reintegration candidates following transformation of fission yeast.

**Gene reintegration strategies**

***rdp1^+^***

JPO-1464 (~307bp upstream of initiation codon) & JPO-1465 (~545bp after termination codon) were used to amplify the *rdp1*^+^ gene along with flanking sequences. This resulted in a PCR product of ~4.5kb with *NotI* and *PstI* restriction enzyme sites at 5’ and 3’ ends respectively. This PCR product was cloned into JP1142 (a modified pR0319 [Adams et al., 2005] from which ARS3002 sequences have been removed by *AatII* digest to generate a *his3^+^* marked integrative vector) to generate JP1250. The restriction enzyme *NheI* (~400bp after termination codon) was used to linearize JP1250 for integration into *rdp1∆* strains. Proper site of integration was checked by PCR analysis and that there was only a single integration was checked by Southern analysis. *SacII* (+3093bp downstream of ATG) and *NcoI* (~700bp after stop codon) were used to digest genomic DNA and following electrophoresis and transfer, the blot was hybridized with a probe derived by PCR from the *rdp1*^+^ ORF using primers JPO-1550 and JPO-1551.

***hrr1^+^***

JPO-1458 (~380bp upstream of initiation codon) and JPO-1459 (~330bp after termination codon) were used to amplify the *hrr1^+^* gene along with flanking sequences. This resulted in a PCR product of ~3.8kb with *NotI* and *PstI* restriction enzyme sites at 5’ and 3’ ends respectively. This PCR product was cloned into JP1142 to generate JP1259. The restriction enzyme *MluI* (with a restriction site ~250bp upstream of initiation codon) was used to linearize JP1259 for reintegration. Proper targeting of the integration was checked by PCR, and that it was a single integration was checked by Southern blotting. *NcoI* (~925bp upstream of initiation codon) and *NheI* (~950bp after ATG) were used to digest genomic DNA and the resulting blot was hybridized with a probe derived by PCR with primers JPO-1479 and JPO-1552 from the *hrr1^+^* ORF.

***cid12^+^***

JPO-1460 (~225bp upstream of initiation codon) & JPO-1461 (~720bp after termination codon) were used to amplify *cid12* gene along with flanking sequences. This resulted in a PCR product of ~2kb with *NotI* and *SalI* restriction enzyme sites at 5’ and 3’ ends respectively. This PCR product was cloned into JP1142 to yield JP1257. The restriction enzyme *PstI* (~540bp after stop codon) was used to linearize JP1257 prior to reintegration. Correct targeting of the integration was verified by PCR, and that a single site of integration had occurred was checked by Southern analysis. *XhoI* (~250bp after ATG) and *NheI* (~1.5kbp after stop) were used to digest genomic DNA and the resulting blot was probed with a *cid12* ORF PCR product of ~275bp generated using primers JPO-1487 and JPO-1556.

***raf1^+^***

*raf1* was amplified in a 3-step PCR reaction. In PCR-1, JPO-1125 (~507bp upstream of start codon with attB sequences) and JPO-1127 with *PstI* (~250bp upstream of start codon) was used to amplify 300bp of *raf1* genomic locus. In PCR-2, JPO-1126 (complementary to JPO-1127) and JPO-1128 (~330bp after stop codon with attB sequences) were used to generate a 3kb Raf1 fragment. In PCR 3, equimolar amounts of PCR products 1 and 2 were used as template for amplification with JPO-1125 and JPO-1128. This resulted in a PCR product of ~3.2kb with attB sequences at 5’ and 3’ ends respectively and a *Pst1* site in the 5’ end of Raf1. This PCR product was then used for a BP recombination reaction with pDONOR 201(Invitrogen). The resulting BP clone (JP1164) was used in the LR recombination reaction with JP1168 (a modified version of JP1142 in which the polylinker sequence between *KpnI* and *SacI* has been replaced with the attB sequence) to generate the *his3^+^* marked *raf1^+^* genomic reintegration vector JP1172. The restriction enzyme *PstI* (with a site ~275bp before start codon) was used to linearize JP1172 for reintegration. Correct targeting of integration was verified by PCR and that a single integration had occurred was verified by Southern analysis. *BamHI* (-750 bp from ATG) and *BstXI* (+1204 from ATG) were used to digest genomic DNA and the resulting blot was probed with a *raf1* ORF PCR product amplified using primers JPO-1146 and JPO-1596.

***raf2^+^***

*raf2* was amplified with JPO-1129 (~507 bp upstream of start codon with attB sequences) and JPO-1130 (~330bp after stop codon with attB sequences). The PCR product was then used for a BP recombination reaction with pDONOR 201. The resulting BP clone (JP1175) was used in the LR recombination reaction with JP1168 to generate the reintegration vector JP1178. The restriction enzyme *Eag I* (with a recognition site 261bp upstream of start codon) was used to linearize JP1178 for reintegration. Correct site of insertion was verified by PCR, and single integration was checked by Southern Blotting. *NdeI* was used to digest genomic DNA and the blotted DNA was probed with a *raf2* ORF PCR product amplified using primers JPO-1595 and JPO-1280.

***rik1***^+^

*rik1* was amplified in a 3-step PCR reaction. In PCR-1, JPO-1164 (~507bp upstream of start codon with attB sequences) and JPO-1166 with *PstI* (~250bp upstream of start codon) were used to amplify 300bp of the *rik1^+^* gene. In PCR-2, JPO-1165 (complementary to JPO-1166) and JPO-1172 (~330bp after stop codon with attB sequences) were used to generate a 4kb *rik1^+^* fragment. In PCR 3, equimolar amounts of PCR products 1 and 2 were used as template for amplification with JPO-1164 and JPO-1172. This resulted in a PCR product of ~4kb with attB sequences at 5’ and 3’ ends respectively and a *PstI* site in the 5’ end of *rik1^+^*. This PCR product was then used for a BP recombination reaction with pDONOR 201. The resulting BP clone (JP1280) was used in the LR recombination reaction with JP1168 to generate the reintegration vector JP1287. The restriction enzyme *PstI* (261bp upstream of start codon) was used to linearize JP1287 for reintegration. Proper targeting was confirmed by PCR, and that only a single integration had occurred was checked by Southern Blotting. *PacI* (~925bp upstream of initiation codon) and *KpnI* (~950bp after ATG) were used to digest genomic DNA and the resulting blot was probed with a *rik1^+^* ORF PCR product of ~ 260bp amplified using primers JPO-1737 and JPO-1729.

***pcu4^+^***

*pcu4* was amplified in a 3-step PCR reaction. In PCR-1, JPO-1155 (~507bp upstream of start codon with *SalI* site) was used with JPO-1119 with *PstI* site (~250bp upstream of start codon) to amplify a short fragment of Pcu4 regulatory sequence. In PCR-2, JPO-1118 (complementary to JPO-1119) and JPO-1156 (~473bp after stop codon with *SpeI* sequences) were used to generate a 3kb *pcu4* fragment. In PCR 3, equimolar amounts of PCR products 1 and 2 were used as template for amplification with JPO-1155 and JPO-1156. This resulted in a PCR product with *SalI* and *SpeI* sequences at 5’ and 3’ ends respectively and a *PstI* site in the 5’ end of *pcu4^+^*. This PCR product was cloned into JP1142 resulting in JP1173. The restriction enzyme *PstI* (~250bp upstream of initiation codon) was used to linearize JP1173 for reintegration. The proper site of integration was checked by PCR, and that integrants were single copy was verified by Southern analysis. Genomic DNA was digested with *XhoI* and *ClaI* and following electrophoresis and blotting, was hybridized with a probe derived from the *pcu4* ORF by PCR with primers JPO-1824 and JPO-1794.

***dcr1^+^***

Oligonucleotides JPO-1056 and JPO-1057 were used to amplify *dcr1^+^* genomic region (~450bp upstream from ATG to +795bp downstream of stop codon), incorporating *SalI* and *SpeI* restriction sites. This PCR fragment was cloned into JP1142 to generate JP 1162. JP 1162 was linearized with *NcoI* for reintegration. Proper integration was checked by PCR, and Southern analysis was used to confirm single copy integration. Genomic DNA was digested with *PflMI* and *BglII* and following electrophoresis and blotting, was hybridized with a probe derived from the *dcr1^+^* ORF by PCR with primers JPO-1597 and JPO-1598.

***clr4^+^***

Reintegration of *clr4^+^* with JP1084 was as described previously (Partridge et al., 2007). Episomal transformation with *clr4^+^* was performed with JP1078 or empty vector JP1045 (Partridge et al., 2007).

***ago1^+^***

Oligonucleotides JPO-1879 and 1880 were used to amplify *ago1^+^* genomic region (~4,050 bp), incorporating *SacI* and *SalI* restriction sites. This PCR fragment was cloned into JP1142 to generate JP1425. JP1425 was linearized with *SpeI* for reintegration. Proper integration was checked by PCR and southern. Genomic DNA was digested with *PvuI* and *XhoI*, and following electrophoresis and blotting was hybridized to a probe derived from *ago1^+^* ORF by PCR with primers JPO-1886 and JPO-433.

**Strain construction**

The *dcr1^+^* gene was reintegrated into PY 3310 and PY 3307 strains.

The *rdp1^+^* gene was reintegrated into PY 4300 and PY 4304 strains.

The *hrr1^+^* gene was reintegrated into PY 4334 and PY 4337 strains.

The *cid12^+^* gene was reintegrated into PY 4338 and PY 4340 strains.

The *raf1^+^* gene was reintegrated into PY 3659 and PY 3518 strains.

The *raf2^+^* gene was reintegrated into PY 3675 and PY 3676 strains.

The *rik1^+^* gene was reintegrated into PY 3776 and PY 3778 strains.

The *pcu4*^+^ gene was reintegrated into PY 5080 and PY 5081 strains.

The *ago1^+^* gene was reintegrated into PY 2211 and PY 5186 strains.

**Chromatin Immunoprecipitation details**

Briefly, 3 x 10^8^ cells were fixed with 3% paraformaldehyde for 18 mins at room temperature, prior to washing with PBS and permeabilization of the cell wall with zymolyase 100T (0.4 mg/ml in PEMS) and incubation at 36^o^C for 20 minutes. Following extensive washing with PEMS, the cell pellet was resuspended in 400ul ChIP lysis buffer and sonicated (3x, 20s each). After pre-clearing with Protein A- agarose beads, lysate was used for immunoprecipitation overnight with each antibody. Antibody-protein complexes were purified using Protein A- agarose beads, washed, and reverse-crosslinking of samples was performed by overnight incubation at 65^0^C in TES, followed by Proteinase K digestion. DNA was purified using the Wizard DNA cleanup kit (Promega) and used for Real-time PCR. Primers used for real time analysis of association with *act1^+^* were JPO-2000 and JPO-2001 (sequences taken from Buhler et al., 2007). Note that for Figure 7D, E and Figure 8, ChIP was performed on cells fixed with 1% paraformaldehyde, and samples were sonicated (3 x 10s each). Additionally, cells were grown in PMG-his media (to maintain episomal plasmid) in Figure 8B.

**Supplementary Reference.**

Adams C, Haldar D, Kamakaka RT (2005) [Construction and characterization of a series of vectors for Schizosaccharomyces pombe](http://www.ncbi.nlm.nih.gov/pubmed/16358314). Yeast 22: 1307-1314.
